# Deletion of B125R increases protection induced by a genotype II African swine fever vaccine candidate

**DOI:** 10.1038/s41541-025-01101-4

**Published:** 2025-03-19

**Authors:** Anusyah Rathakrishnan, Ana Luisa Reis, Katy Moffat, Lynnette Goatley, Elisenda Viaplana, Jose Carlos Mancera, Alicia Urniza, Linda K. Dixon

**Affiliations:** 1 The Pirbright Institute, Surrey, England UK; 2 Zoetis Manufacturing and Research Spain, Finca la Riba, L’Hostalnou de Bianya, Girona, Spain; 3 Zoetis Belgium, Zaventem, Belgium

**Keywords:** Immunology, Microbiology

## Abstract

A modified live attenuated African swine fever genotype II virus, GΔDKE-CmutQ96R/K108D, with deletions of three genes, *DP148R*, *EP153R,* and *K145R* and expressing a mutated CD2v protein with a non-haemadsorbing phenotype, was further modified by first removing two reporter gene cassettes expressing fluorescent proteins. The *B125R* gene was then deleted and one reporter cassette was reinserted as a marker. Groups of pigs were immunised with this virus using a range of doses from 100 to 10,000 infectious particles. One pig immunised with the lowest dose reached a moderate severity humane endpoint. The other pigs showed mild or no clinical signs. Low levels of the virus used for immunisation were detected post-immunisation. After challenge with virulent virus, all pigs were protected, and few clinical signs were observed. Low levels of replication of the challenge virus were detected in seven from the twenty-three challenged pigs and no virus in the remaining pigs.

## Introduction

African swine fever virus (ASFV) causes a hemorrhagic fever in domestic pigs and wild boar, which can result in a case fatality rate approaching 100%. The disease has a very high socio-economic impact and threatens global food security. At least 23 genotypes, based on partial sequencing of the B646L major capsid protein gene, are circulating in eastern Africa, but only genotypes I and II have spread outside Africa^1–3^. Genotype I was introduced to Portugal in 1957 and 1960 and eradicated except for Sardinia by 1999. Genotype II was introduced to Georgia in the Caucasus in 2007 and spread to Russia and Eastern Europe entering the EU in 2014. In 2018, genotype II virus was introduced and spread rapidly through China (WOAH WAHIS, FAO Empres)^1,4^ and to almost all Asian countries (WOAH WAHIS, FAO Empres)^1,4^. Attenuated genotype I ASFV^5^ and virulent recombinant genotypes I and II hybrid viruses have also been reported in China and Vietnam^6,7^. Modified live ASFV vaccines (MLVs) were licensed for use in Vietnam in 2023 but are not widely available^8^.

ASFV is a large, cytoplasmic DNA virus containing up to 190 open reading frames (ORFs). The multi-layered virus particle contains about 70 proteins^9^. Many virus ORFs are not required for replication in cells and code for proteins which evade the host innate immune response including the type I interferon response and cell death^10^.

MLV candidates have been constructed by deleting different combinations of genes to attenuate viruses in pigs and induce a protective immune response^11–13^. We previously described a genotype II ASFV MLV candidate, in which three genes (*K145R*, *EP153R,* and *DP148R*) were deleted and *EP402R*, which codes for the CD2v protein, was modified at 2 amino acids to make the virus non-haemadsorbing (non-HAD)^14^. When pigs were immunised and boosted with this MLV, GΔDKE-CmutQ96R/K108D, we obtained dose-dependent efficacies (83–100%) against homologous genotype II virulent virus challenge (Georgia 2007/1). In one experiment, one pig reached the moderate humane severity endpoint at 7 days post-immunisation (dpi) (Rathakrishnan et al., unpublished). We, therefore, planned further modifications to this virus to improve the safety and maintain or improve efficacy against challenge. To do this, we first removed the fluorescent protein gene cassettes used as markers to select specific gene deletions so that these could be reused to select additional gene deletions. The *B125R* gene was then deleted since we identified this protein as a possible serology marker to distinguish infected from vaccinated animals (DIVA). Replication kinetics of this virus, GΔBDKE-CmutQ96R/K108D from which the *B125R* gene was deleted, in porcine macrophages was similar to the wildtype and parental gene-modified viruses. However, the final virus titre was approximately 10 times lower. Pigs were immunised with a range of doses and challenged with virulent genotype II Georgia 2007/1. The results showed that this virus induced higher levels of protection against the virulent genotype II virus across a wide range of doses compared to the parental virus since all 23 of the challenged pigs survived. The potential to use B125R as a serological DIVA marker was evaluated by screening sera from pigs immunised with attenuated ASFV that express B125R protein. The results indicated 50–75% of sera were positive for B125R. Thus, additional DIVA targets are likely to be required for use in the field.

## Results

### Identification of B125R as an immunogenic protein

**Fig. 1:**
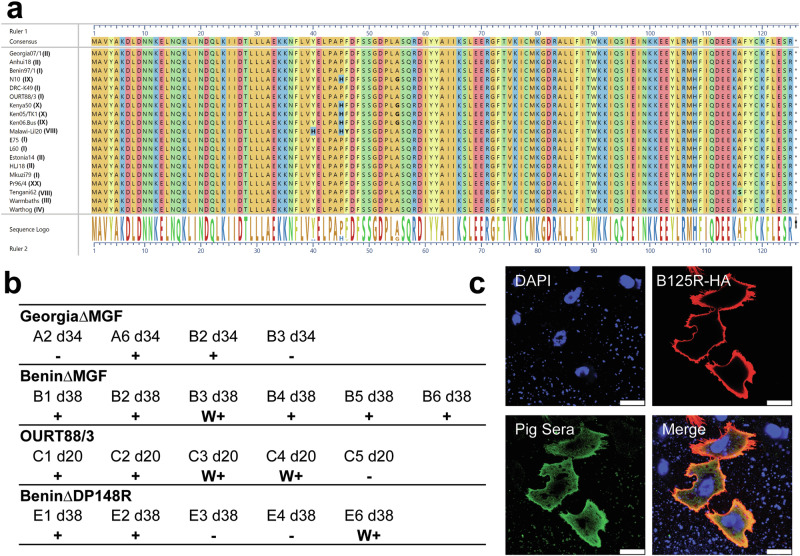
Characteristics of ASFV B125R protein. **a** Amino acid sequences of B125R from 19 ASFV isolates were compared using Clustal Omega, on MegAlign Pro (DNASTAR Lasergene 17). The ASFV genotypes are given beside the names of the isolates in brackets. Amino acid residues that differ are shown in bold and highlighted in a different colour. **b, c** Vero cells were transfected with a plasmid expressing B125R fused to an HA epitope tag at the C-terminus. The cells were fixed and permeabilised then immunostained with anti-HA antibody or pre-challenge pig sera from immunised pigs. Following secondary antibody staining with goat anti-mouse Alexa Fluor 568 or goat anti-pig Alexa Fluor 488, cells were counterstained with DAPI. **b** indicates which of the pre-challenge sera from pigs immunised with GeorgiaΔMGF, BeninΔMGF, OURT88/3, or BeninΔDP148R reacted with the transiently expressed B125R protein. A negative symbol **–** indicates no staining of B125R protein was detected, **W+** indicates that low levels of B125R staining by antibodies were detected and + indicates that strong B125R staining by antibodies was detected in sera tested. An example of a vaccinated pig serum binding to transiently expressed B125R protein is shown in (**c**). In this staining of the nucleus via DAPI is shown in blue, the red shows staining of the expressed B125R protein by anti-HA and green shows the binding of B125R antibodies by pre-challenge serum from a vaccinated pig. The images are shown at a magnification of 63×, where the scale bar represents 25 µm. The detailed images of the individual pig serum tested are shown in Supplementary Fig. 1.

Non-essential ASFV genes that express immunogenic proteins are potential markers to DIVA by serology. To identify candidate DIVA markers, we expressed individual ASFV proteins by transfecting plasmids expressing the genes of interest, under the control of a eucaryotic promoter into Vero cells. The fixed and permeabilised cells were stained with sera from previous experiments in which the pigs were immunised with different attenuated gene-deleted ASFV viruses. The pre-immune and pre-challenge sera used were from pigs immunised with attenuated viruses GeorgiaΔMGF^15^, BeninΔMGF^16^, OURT88/3^16^, or BeninΔDP148R^17^ (Fig. 1b, Supplementary fig. 1). This screen identified B125R as an immunogenic protein. The ASFV *B125R* gene (FR682468.2: nt 106557 to 106394) codes for a poorly characterised protein. The protein sequence is highly conserved among different ASFV isolates (Fig. 1a). At least 75% of the 20-pre-challenge sera screened detected the transiently expressed B125R protein by immunofluorescence in Vero cells (Fig. 1b, c, and Supplementary fig. 1). Interestingly, the B125R protein was localised close to the plasma membrane. Since B125R does not have a predicted transmembrane domain (TMHMM 2.0, Phobius), its localisation is likely to involve interaction with other proteins (Fig. 1c, top right panel).

### Deleting the B125R gene from the modified attenuated ASFV GΔDKE-CmutQ96R/K108D strain: replication kinetics and whole genome sequencing

**Fig. 2:**
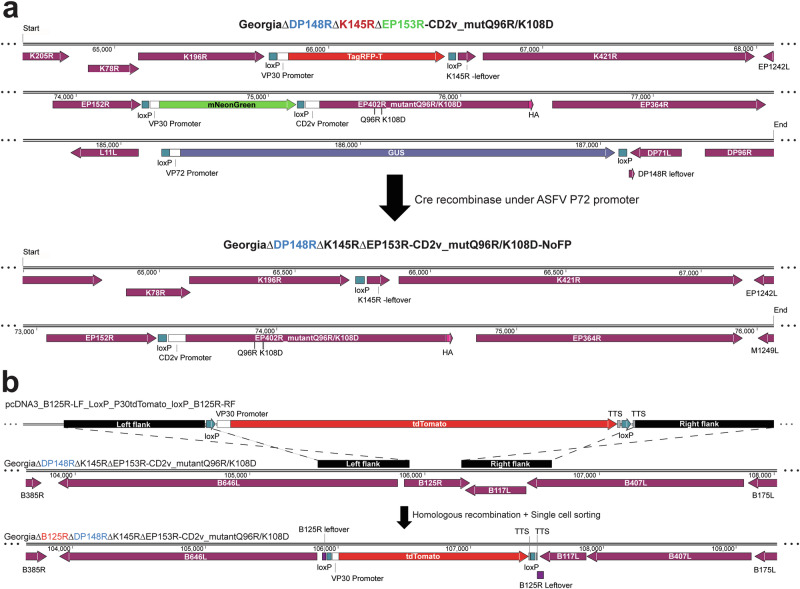
The construction of recombinant ASFV virus—GeorgiaΔBDKE-CmutQ96R/K108D. **a** Before deleting *B125R* from the parental virus GeorgiaΔDP148RΔK145RΔEP153R-CD2v_mutQ96R/K108D (here designated GeorgiaΔBDKE-CmutQ96R/K108D), the existing fluorescent reporter gene markers flanked by loxP (TagRFP-T, mNeonGreen were removed (top panel). PBMs were infected with the parental virus and then transfected with a plasmid expressing Cre recombinase. After two days, cells not expressing any fluorescent markers were bulk sorted via FACS into complete RPMI and these cells were subjected to two rounds of limiting dilutions. The final selected virus, GeorgiaΔDP148RΔK145RΔEP153R-CD2v_mutQ96R/K108D-NoFP, which was non-haemadsorbing and non-fluorescent, but still containing the GUS marker was grown and titrated before being used as template to delete the *B125R* gene (bottom panel). **b** Using the newly produced, GeorgiaΔDP148RΔK145RΔEP153R-CD2v_mutQ96R/K108D-NoFP as parental virus, the *B125R* gene was deleted from nucleotides 106,585–106,886 (ASFV Georgia 2007/1: FR682468.2). The transfer plasmid, pΔB125R-tdT, contains left and right flanking regions of the *B125R* gene, either side of the fluorescent marker tdTomato, under the control of ASFV VP30 promoter. Purified PBMs were infected with GeorgiaΔDP148RΔK145RΔEP153R-CD2v_mutQ96R/K108D-NoFP, then transfected with pΔB125R-tdT. After 36–48 post-infection-transfection, recombinants expressing tdTomato^+^ were isolated by single-cell sorting into PBMs using FACS. Following subsequent rounds of single-cell isolation and limiting dilutions, GeorgiaΔBDKE-CmutQ96R/K108D was obtained.

Our previous experiments showed that one pig immunised with the GΔDKE-CmutQ96R/K108D ASFV strain reached a moderate severity humane endpoint (Rathakrishnan et al., unpublished). To improve the safety of this virus we planned to delete in addition the *B125R* gene and test the replication kinetics in cells and the safety and induction of protection against challenge in pigs. The parental GΔDKE-CmutQ96R/K108D recombinant virus contains three reporter proteins, including beta-glucuronidase (β-GUS) and 2 fluorescent markers, TagRFP-T, and mNeonGreen. To delete the *B125R* gene from this virus strain, we first deleted all fluorescent reporter genes so that one of these could be used again to isolate a virus with an additional deletion of the *B125R* gene. Cre recombinase was transiently expressed in infected cells to remove the reporter using the loxP sites flanking the reporter cassettes (Fig. 2a)^18^. Infected cells that were non-fluorescent were obtained by bulk cell FACS sorting. As the virus was non-HAD and now non-fluorescent, we identified those wells that contained cells showing cytopathic effects (CPEs), followed by detection of the ASFV *p72* gene by PCR to confirm the infection. Following a second round of limiting dilution, all expected genes (*DP148R*, *K145R*, *EP153R*, *TagRFP-T,* and *mNeonGreen*) except the β-GUS reporter gene were confirmed to be absent by PCR analysis.

The next step was to delete the *B125R* gene from this non-fluorescent virus GΔBDKE-CmutQ96R/K108D using a fluorescent tdTomato fluorescent protein as a reporter. The B125R gene is located between two known essential genes, *B646L* which codes for the ASFV major capsid protein p72 and the *B117L* gene encoding the integral membrane protein B117L, recently characterised as having low pH membrane permeabilizing activity^19^ (Fig. 2b). To avoid disrupting transcription signals or sequences for these essential genes, 27 bp of *B125R* at the 5′ end was left intact (Fig. 2b and Supplementary fig. 2). The 3′ end of *B117L*, is read toward the left of the genome and overlaps with the 3′ end of *B125R*, which is read towards the right of the genome; hence, 49 bp at the 3′ end of *B125R* was left intact (Fig. 2b and Supplementary Fig. 2). To avoid transcription read through from the reporter cassette into downstream genes, including the *B117L* gene, an ASFV transcription termination signal (TTS)^20^ consisting of a stretch of 10 poly-T nucleotides was included at the 3′ end of the reporter gene cassette. To avoid read-through in the opposite direction from the *B117L* gene into the reporter cassette, a TTS was included on the opposite DNA downstream from the *B117L* gene. PBM cells were infected with the newly produced non-HAD, non-fluorescent parental virus and transfected with the transfer plasmid pΔB125R-tdTomato. The recombinant virus GΔBDKE-CmutQ96R/K108D with *B125R* deleted and expressing tdTomato protein was isolated by a combination of single-cell sorting and limiting dilutions^21^. The correct insertion of reporters and *B125R* gene deletion was confirmed by PCR and sequencing (see Supplementary Fig. 2).

**Fig. 3:**
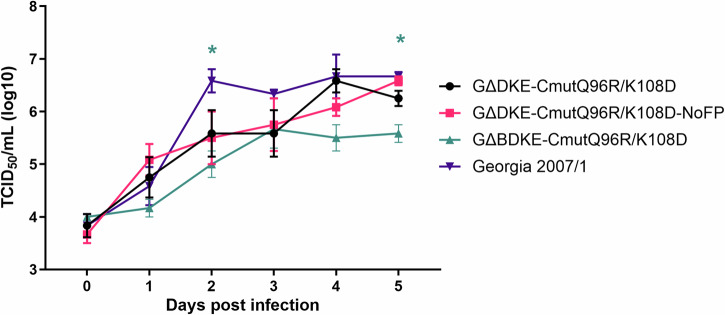
Multistep growth curve of recombinant viruses compared to Georgia 2007/1. Purified PBMs were infected with the following viruses: (i) Georgia 2007/1, (ii) GeorgiaΔDP148RΔK145RΔEP153R-CD2v_mutQ96R/K108D (GΔDKE-CmutQ96R/K108D), the previous vaccine virus, (iii) GeorgiaΔDP148RΔK145RΔEP153R-CD2v_mutQ96R/K108D- NoFP (GΔDKE-CmutQ96R/K108D-NoFP), the previous vaccine virus after removing the fluorescent reporter markers, *TagRFP-T* and *mNeonGreen*, and (iv) GeorgiaΔB125RΔDP148RΔK145RΔEP153R-CD2v_mutQ96R/K108D (GΔBDKE-CmutQ96R/K108D), the current vaccine candidate with *B125R* gene deleted, at MOI 0.01 in triplicates. Viruses were harvested from both cells and supernatants at different time points and titrated on PBMs in quadruplicates. Multistep growth curves of the gene-deleted viruses and wild-type Georgia 2007/1 are shown over the course of 5 days, where Day 0 represents the inoculum (x-axis). Virus titres are shown on a log 10 scale on the y-axis as TCID50/mL. Two-way ANOVA with Dunnett’s multiple comparisons test was performed to evaluate the differences between the recombinant viruses and the wildtype virus. Significant differences are represented by asterisk where * is *p* < 0.05; the colour of the asterisk represents the respective virus.

Whole genome sequencing of GΔBDKE-CmutQ96R/K108D virus confirmed (i) the removal of the previous fluorescent reporter markers, TagRFP-T and mNeonGreen, (ii) the deletion of *B125R*, in addition to deletion of *K145R*, *EP153R,* and *DP148R*, (iii) the insertion of reporter gene, tdTomato, and the presence of the GUS gene, and (iv) no additional changes in the CD2v protein, except for the changes made previously, Q at position 96 to R and K at position 108 to D (Supplementary Table 1). No additional changes were observed in the genomes of the recombinant viruses compared to the parental Georgia 2007/1 genome. The GΔBDKE-CmutQ96R/K108D virus was non-HAD as expected and replicated to titres approximately a log lower, compared to the other parental recombinant viruses and Georgia 2007/1 wildtype virus. This trend was observed from 1 dpi and reached low significance (*p* < 0.5) at 2 dpi and 5 dpi (Fig. 3).

### Immunisation of pigs with GΔBDKE-CmutQ96R/K108D at different doses and with or without boost induces few clinical signs

Aliquots of GΔBDKE-CmutQ96R/K108D were grown for two passages in a proprietary cell line (subject to patent restrictions) and supplied ready to use by Zoetis. An adaptation of the ASFV MLV to the cells was not required and the virus phenotype and virus titres obtained were similar to those obtained in primary macrophages. The whole genome sequence of the GΔBDKE-CmutQ96R/K108D virus grown for two passages in the cell line was not determined. Our experience is that major genome changes are very unlikely during this low number of passages since an adaptation to the cells was not required and no phenotypic changes were detected. Viruses were titrated on PBM-derived cells before being used in the immunisation studies.

**Fig. 4:**
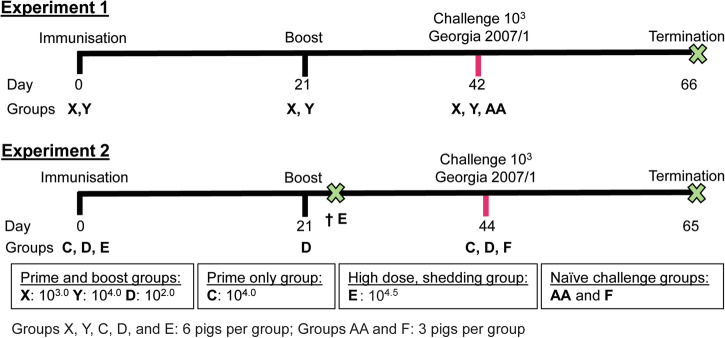
Experimental timeline. The timeline for the 2 pig immunisation experiments is shown. Groups X, Y and D were primed and boosted with GeorgiaΔBDKE-CmutQ96R/K108D at different doses, 10^3^, 10^4^, and 10^2^ TCID_50_ in 1 mL PBS or L2-RPMI intramuscularly, respectively. Pigs in Group C were immunised with 10^4^ TCID_50_ without a booster dose. Two groups of non-immune pigs (Groups AA and F) were challenged in parallel to the immunised pigs (Groups X, Y, D, and C). In experiment 2, pigs in Group E were immunised at 10^4.5^ TCID_50_ and were culled at 23 dpi, where † denotes the culling of Group E.

**Fig. 5:**
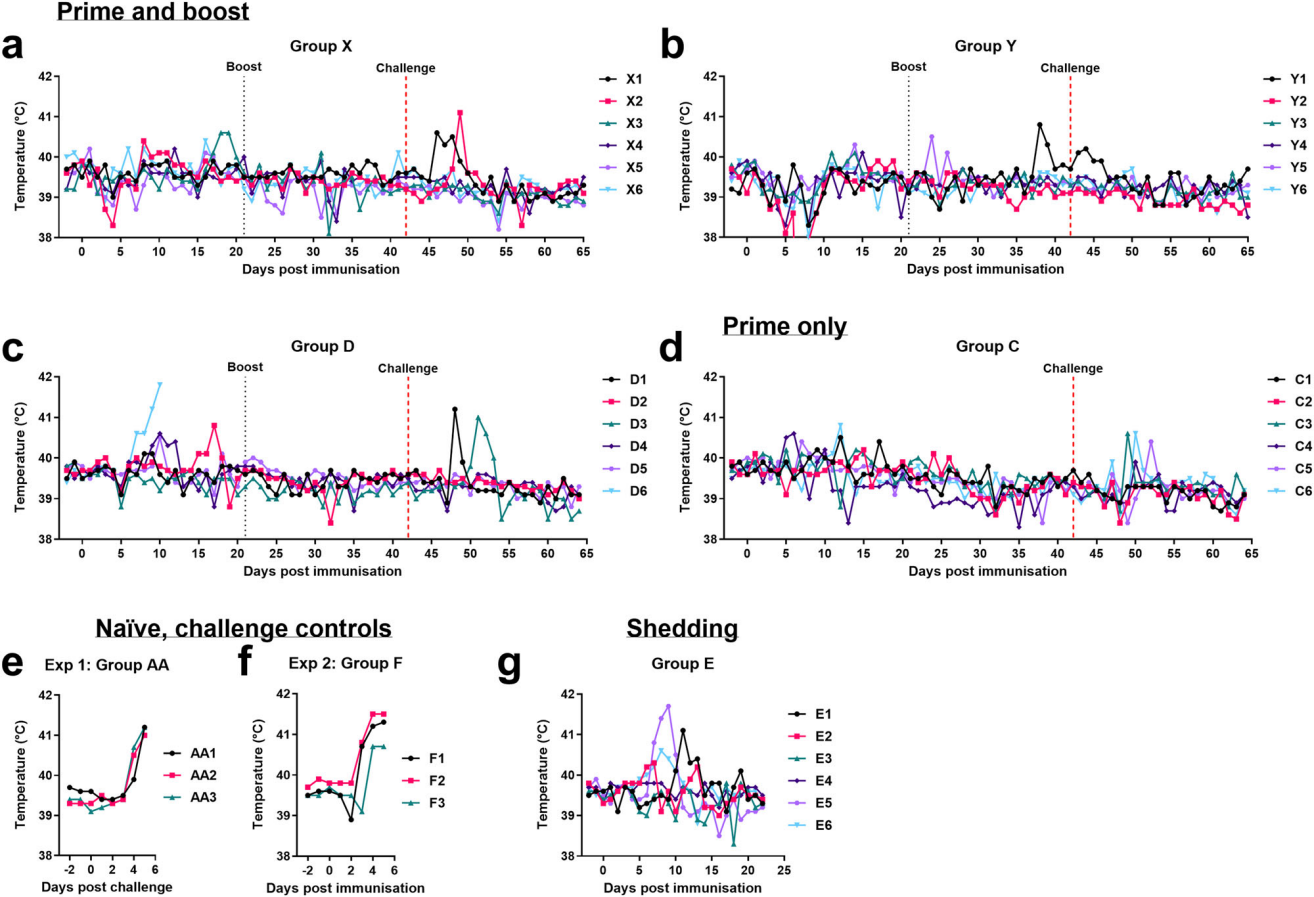
Daily temperatures of pigs. Rectal temperatures were recorded daily for pigs immunised and boosted with GΔBDKE-CmutQ96R/K108D at different doses **a** 10^3^ (Group X). **b** 10^4^ (Group Y). **c** 10^2^ (Group D). **d** Pigs immunised with 10^4^ without a booster dose (Group C). The temperatures for the non-immune, challenge control groups were recorded following challenge with Georgia 2007/1 isolate, for (**e**) experiment 1 and **f** experiment 2. **g** pigs immunised with 10^4.5^ TCID_50_ dose (Group E).

**Fig. 6:**
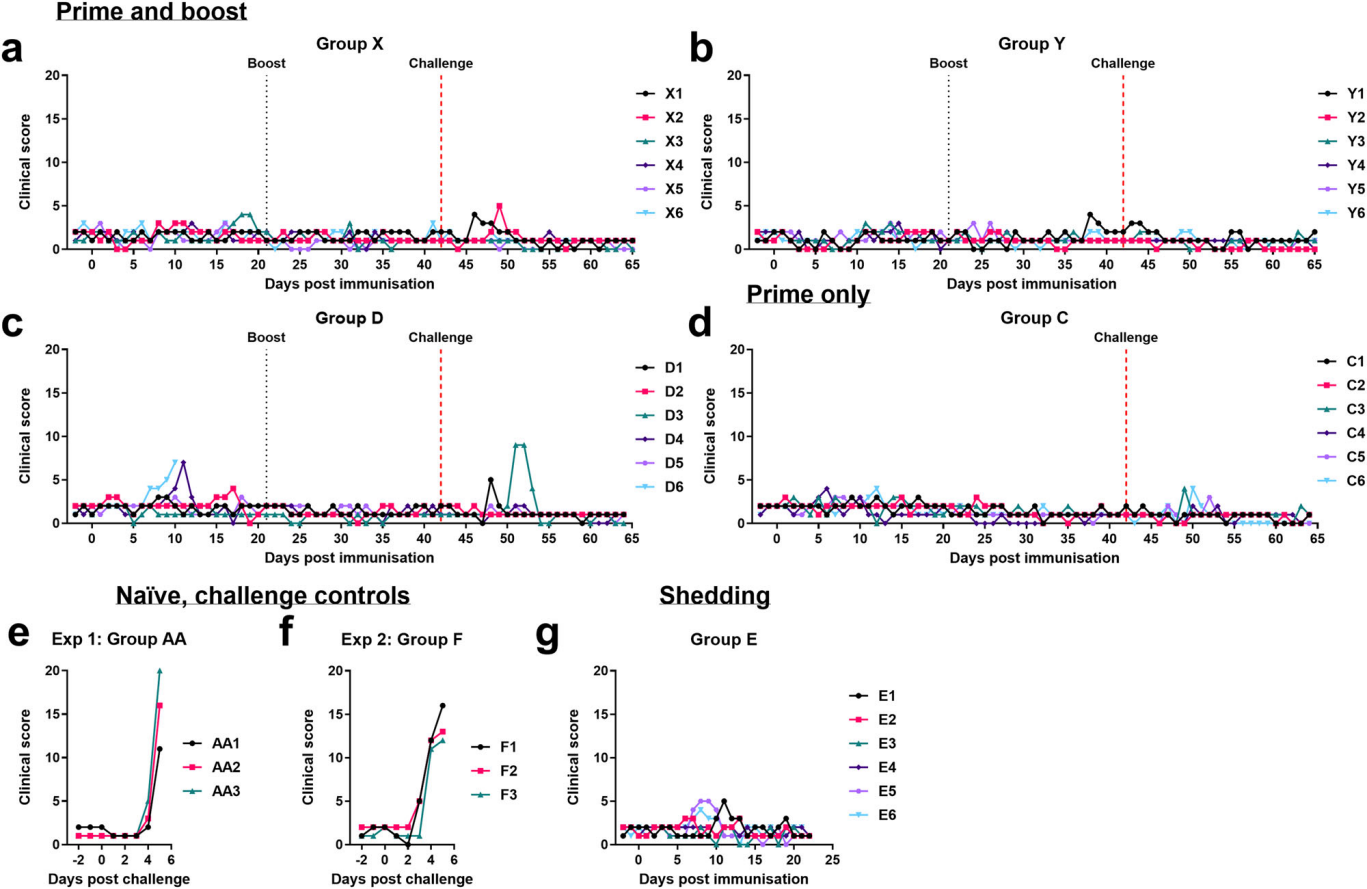
Daily clinical scores of pigs. Cumulative clinical scores based on clinical signs observed daily for pigs immunised and boosted with GΔBDKE-CmutQ96R/K108D at different doses **a** 10^3^ (Group X), **b** 10^4^ (Group Y), and **c** 10^2^ (Group D). **d** Pigs immunised (10^4^) without a booster dose (Group C) and The clinical scores for the non-immune, challenge control groups were recorded following challenge with Georgia 2007/1 isolate, for (**e**) experiment 1 and (**f**) experiment 2. **g** Pigs immunised with 10^4.5^ TCID_50_ dose (Group E).

In Experiment 1, 2 groups of six female Large White Landrace cross pigs were immunised with 10^3^ or 10^4^ TCID_50_ GΔBDKE-CmutQ96R/K108D in 1 mL of PBS intramuscularly, respectively (Fig. 4). Both groups were boosted by the same route and with the same vaccine doses at 21 dpi. In each group, one pig had a temperature above 40.5 °C. Pig X3 immunised with 10^3^ TCID_50_ GΔBDKE-CmutQ96R/K108D had 2 days of increased temperatures at 18–19 dpi (Fig. 5a), while pig Y1 (10^4^ TCID_50_) had a single day of increased temperatures at 38 dpi (Fig. 5b). No other clinical signs were observed in either group before challenge (Fig. 6a, b).

To evaluate the lowest dose which could induce protection, in Experiment 2, a group of six pigs (Group D) was immunised and boosted with a tenfold lower dose, 10^2^ TCID_50_, of GΔBDKE-CmutQ96R/K108D (Fig. 4) in 1 mL L2 stabiliser in RPMI. This allowed us to also evaluate the impacts of the stabiliser on clinical signs and induction of protection.

After immunisation, three pigs of Group D, D2, D4, and D6, had temperatures above 40.5 °C. Pigs D2 and D4 had a single day of fever, on dpi 10 and 17, respectively (Figs. 5c and 6c). Pig D6 had 4 days of temperatures above 40.5 °C, starting from 7 dpi, and on the fourth day of elevated temperature, showed reduced eating and was lethargic (Figs. 5c, 6c) and was culled after reaching the moderate humane endpoint at 10 dpi.

According to the VICH Guideline for Animal Safety for Veterinary Live and Inactivated Vaccines (Doc. Ref. EMEA/CVMP/VICH/359665/2005), a one-dose (just prime vaccination) or two-dose (prime and boost), testing of the vaccine candidates should be carried out in the maximum release potency. Therefore, a group of six pigs (Group C) were immunised with 10^4^ TCID_50_ of GΔBDKE-CmutQ96R/K108D in 1 mL of L2-RPMI, with no booster dose (Fig. 4). This dose was chosen based on Experiment 1, where both 10^3^ and 10^4^ doses were equally safe and induced 100% protection. This group of pigs was immunised in parallel with Group D. Only two pigs, C4 and C6, had temperatures above 40.5 °C for a single day, at 6 and 12 dpi, respectively (Fig. 5d). No other clinical signs were observed in any of the pigs (Fig. 6d).

### GΔBDKE-CmutQ96R/K108D-immunised pigs are effectively protected against the virulent ASFV Georgia 2007/1 whether given a single dose or repeat doses

The immunised pigs in Groups X (10^3^) and Y (10^4^) were challenged at 42 dpi in parallel with a group of three naïve pigs (Group AA) (Fig. 4). In Experiment 2, the pigs in Group D (10^2^) and C (10^4^, prime only) were challenged at 44 dpi, in parallel with the non-immunised pigs of Group F (Fig. 4). All the pigs were challenged intramuscularly with 10^3^ HAD_50_ of the ASFV Georgia 2007/1 isolate in 1 mL PBS.

The immunised pigs in all four groups, regardless of dose and regimen used, were 100% protected against the virulent Georgia 2007/1 isolate and survived until the end of the experiment. In contrast, both groups of the naïve non-immunised pigs developed temperatures for at least 2 days, before they stopped eating and became very lethargic (Figs. 5e, f, 6e, f). All six of the naïve pigs were culled at 5 days post-challenge (DPC) at the defined moderate severity humane endpoint.

Two of the pigs in Group D that received the lowest dose of vaccine had clinical signs after challenge (Figs. 5c and 6c). Pig D1 had a single day of temperature >40.5 °C and was lethargic at 4 dpc. Pig D3 had 2 days of raised temperatures, was picking at food and lethargic at 7–8 dpc. Both recovered without further signs until the end of the study. Only two pigs in Group X that were immunised with 10^3^ TCID_50_/mL of GΔBDKE-CmutQ96R/K108D had temperatures of >40.5 °C for a single day. Pig X1 at 4 dpc and pig X2 at 7 dpc (Fig. 5a). No other clinical signs were observed in this group until the end of the experiment (Fig. 6a).

None of the Group Y pigs that were primed and then boosted with 10^4^ TCID_50_ of GΔBDKE-CmutQ96R/K108D developed any clinical signs after challenge (Figs. 5b and 6b). In contrast, two pigs from Group C which were primed with the same dose without a booster dose had temperatures >40.5 °C after challenge with ASFV Georgia 2007/1. Both pigs had a single day of elevated temperature, pig C3 at 5 dpc and pig C6 at 6 dpc (Fig. 5d).

### Mild gross lesions were observed in the GΔBDKE-CmutQ96R/K108D-immunised pigs that were challenged with the ASFV Georgia 2007/1

**Fig. 7:**
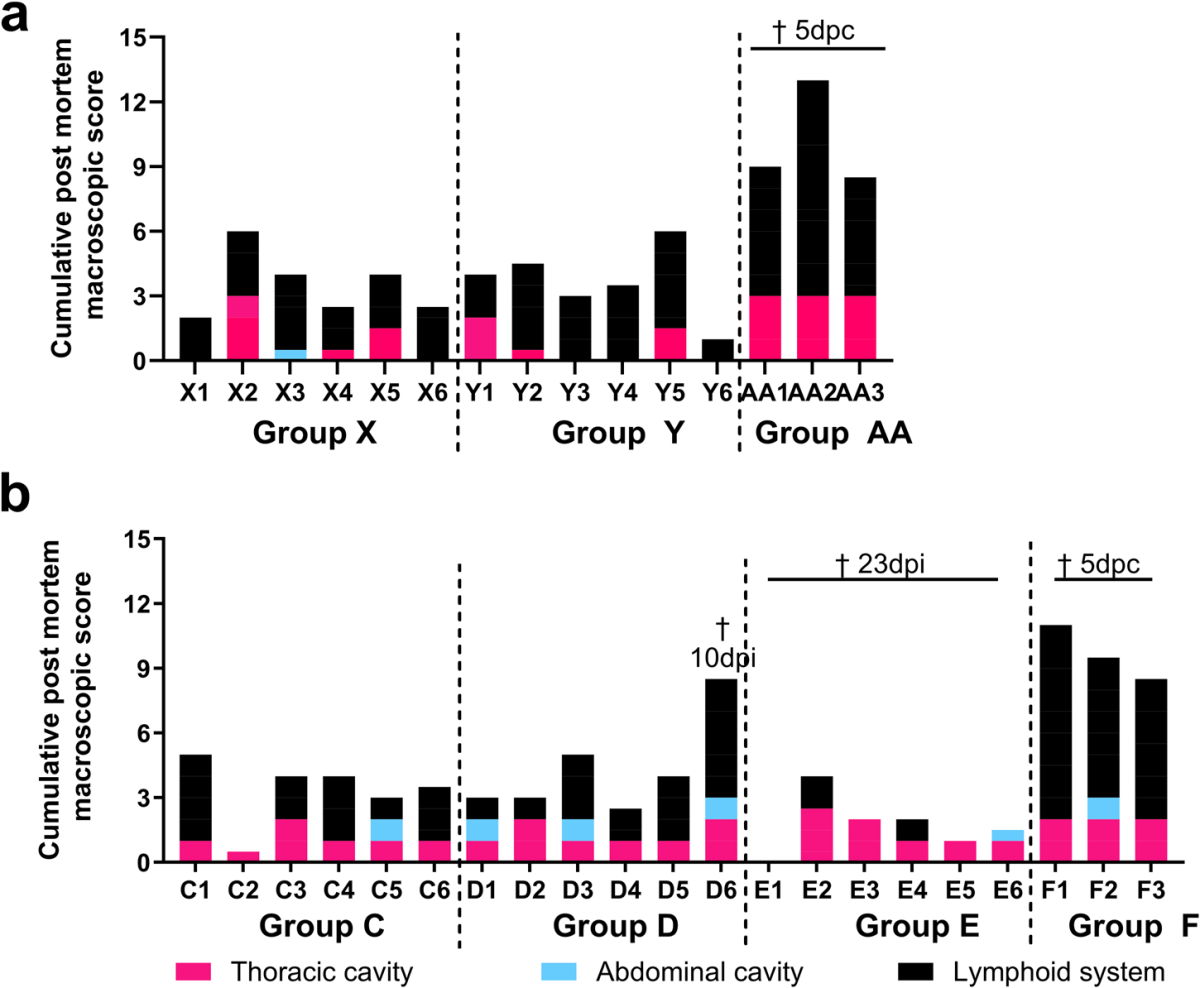
Post-mortem macroscopic lesion scoring. Lesions observed were scored and displayed as a cumulative score observed in different organs of the pig (different coloured bars). Lesions in the thoracic cavity include the presence of thoracic exudates as well as lesions affecting cardio-respiratory system (pink bar). Lesions in the abdominal cavity include the presence of ascites along with the presence of lesions affecting the gastrointestinal system including the stomach, intestines, liver and gallbladder (blue bar). Lesions in lymphoid tissues including tonsils, thymus, spleen and several lymph nodes are depicted by the black bar. Results for individual pigs in different groups as labelled on the x-axis are shown in (**a**) for experiment 1; and (**b**) for experiment 2. † above the individual bars or groups denotes the days on which the individual pigs or groups of pigs were culled. Days post-immunisation are indicated as dpi and days post-challenge as DPC.

Gross lesions typical of acute ASFV infections were observed at 5 dpc during post-mortems of non-immune challenge control pigs (Group AA and F) (Fig. 7a, b). These included mild to moderate enlargement of lymph nodes with or without haemorrhages, hyperaemic splenomegaly, erythematic tonsils, presence of hydropericardium and ascites, as well as mild to moderate levels of non-collapsed lungs, with distention of the interlobular septa. In one pig (F1), oedema was observed around the kidneys. Some lesions were observed in pig D6 which was culled at 10 dpi after immunisation with 10^2^ TCID_50_ of GΔBDKE-CmutQ96R/K108D, although most of the lesions were milder than many of the challenge controls (Fig. 7a).

The post-mortems of surviving pigs that were terminated at the end of the experiment (>20 DPC), revealed fewer lesions overall in immunised pigs regardless of doses and regimen. The presence of mild hydropericardium and mild ascites, along with mild enlargement of spleens and lymph nodes were noted in the immunised pigs after the challenge. Four out of the 23 pigs (X5, Y5, C2, and C3) that survived challenge had mildly non-collapsed lungs. Two pigs (X2 and Y1) had focal areas of lung consolidation. Pig X2 also had patches of haemorrhages across the lung parenchyma. Five pigs (D5, X3, X5, Y2, and Y5) had fibrinous adhesions between the pericardium and the myocardium. This has been described in pigs infected with ASFV strains of varied virulence^22–25^. However, it is difficult to determine if ASFV and/or concomitant bacterial infections led to this manifestation.

### Mild to moderate levels of ASFV were detected in the GΔBDKE-CmutQ96R/K108D-vaccinated pigs after immunisation and challenge

**Fig. 8:**
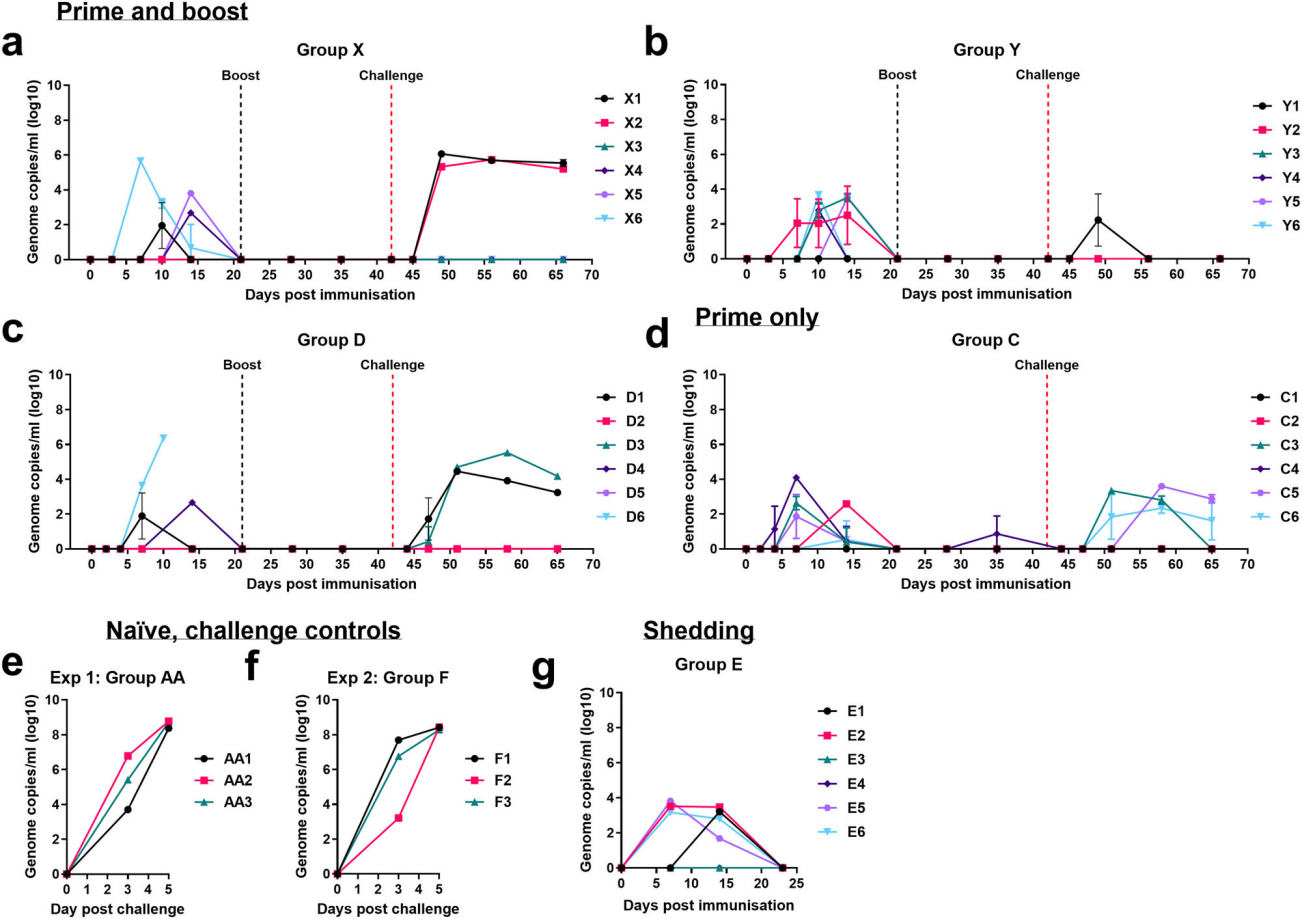
Levels of ASFV genome in whole blood from pigs. Viral genome copies per mL blood were determined by qPCR for pigs immunised and boosted with GΔBDKE-CmutQ96R/K108D at different doses **a** 10^3^ (Group X), **b** 10^4^ (Group Y), and **c** 10^2^ (Group D), **d** pigs immunised (10^4^) without a booster dose (Group C). Viremia for the non-immune, challenge control groups were measured following challenge with Georgia 2007/1 isolate, for **e** experiment 1 and **f** experiment 2. The cut-off for accurate detection is 10^2^ ASFV genome copies/mL; below this, the exact genome copy cannot be determined. Any value below this indicates the presence of ASFV genome but the exact titres cannot be determined. **g** Pigs immunised with 10^4.5^ TCID_50_ dose (Group E).

**Fig. 9:**
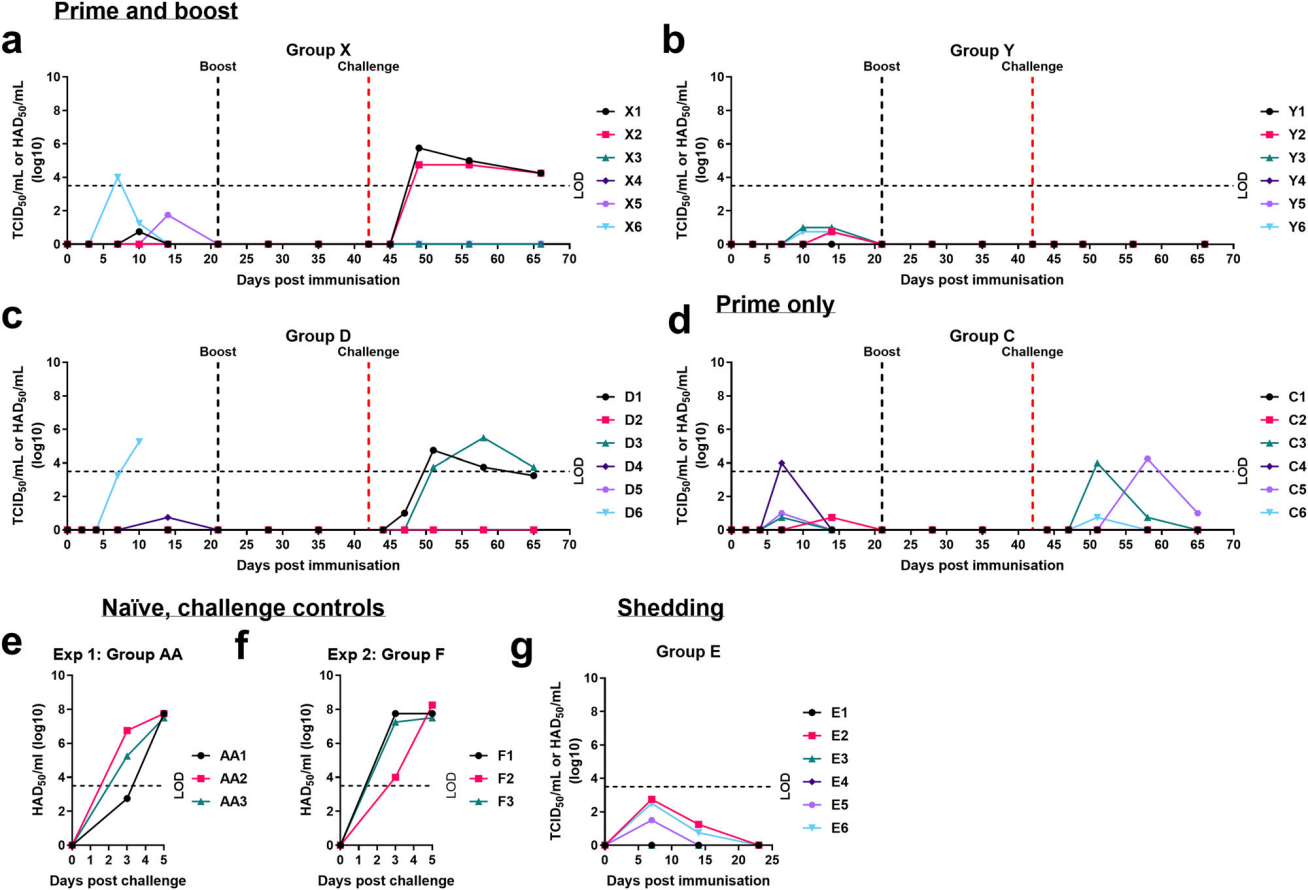
Levels of infectious ASFV in whole blood from pigs. Levels of viremia were determined by titrating virus in PBMs from the whole blood of pigs immunised and boosted with GΔBDKE-CmutQ96R/K108D at different doses **a** 10^3^ (Group X), **b** 10^4^ (Group Y), and **c** 10^2^ (Group D), **d** pigs immunised (10^4^)without a booster dose (Group C) and **g** pigs immunised with 10^4.5^ TCID_50_ dose (Group E). Viremia for the non-immune, challenge control groups was measured following challenge with Georgia 2007/1 isolate. Results for the control Group AA in experiment 1 are shown in (**e**) and Group F for experiment 2 in (**f**). The dashed line on the y-axis represents the cut-off below which accurate measurement of infectious virus per mL blood was not obtained. Any value below this indicates the isolation of infectious ASFV but the exact titres cannot be determined. Results for Group E were below this level (**g**).

Higher levels of the vaccine virus (10^5.3^ TCID_50_/mL, 10^6.4^ genome copies/mL) were detected in blood from pig D6 on 10 dpi when it was culled at the moderate severity humane endpoint (Figs. 8c and 9c). The virus isolated from this pig remained non-HAD and still expressed the tdTomato protein. Only 1 other pig (X6) had moderate levels of ASFV genome detected (10^5.7^) at 7 dpi and these then reduced to non-detectable levels at 21 dpi (Fig. 8a). The amount of infectious virus detected from this pig was 10^3.8^ TCID_50_/mL at 7 dpi, which reduced to non-detectable levels at 14 dpi (Fig. 9c). The remainder of the immunised pigs had low levels of both vaccine virus genome (10^1.9–4.1^ genome copies/mL) and infectious virus (10^0.8–4.0^ TCID_50_/mL in blood (Figs. 8a–d and 9a–d). By 21 dpi, vaccine virus DNA was no longer detected in any pigs, except for pig C4, which had genome levels below the accurate detection limit at 35 dpi.

After challenge, the non-immune challenge control pigs all had >10^8.0^ genome copies/mL (>10^7.5^ TCID_50_/mL) in blood at 5 dpc, the day they were culled (Figs. 8e, f, 9e, f). In contrast lower levels of ASFV genome and infectious particles were detected in two pigs from Group D (Figs. 8c, 9c), in two pigs from Group X (Figs. 8a, 9a) and in three pigs from Group C (Figs. 8d, 9d). Only one pig from Group Y (Y1) had detectable AFSV genome after challenge (Figs. 8b, 9b). In three pigs from Group C, which received the same amount of the vaccine virus as Group Y, but without a boost, virus genome was detected, at low levels (10^2.3–3.6^ genome copies/mL blood). Pigs X1 and X2, which received 10^3^ TCID_50_ vaccine had 10^5.7–6.1^ maximum genome copies/mL at 7 dpc and 14 dpc, respectively. The pig D1 immunised with 10^2^ TCID_50_, had a maximum of 10^4.5^ at 7 dpc, while pig D3 had 10^5.5^ genome copies/mL blood at 14 dpc. Both pigs showed decreasing levels of ASFV in blood at the end of the experiment. None of the infectious viruses detected after challenge in any of the groups of immunised pigs expressed fluorescent proteins and all were HAD as expected for the challenge virus.

### Early antibody and cellular responses detected in the pigs immunised with the GΔBDKE-CmutQ96R/K108D regardless of doses and immunisation regime

**Fig. 10:**
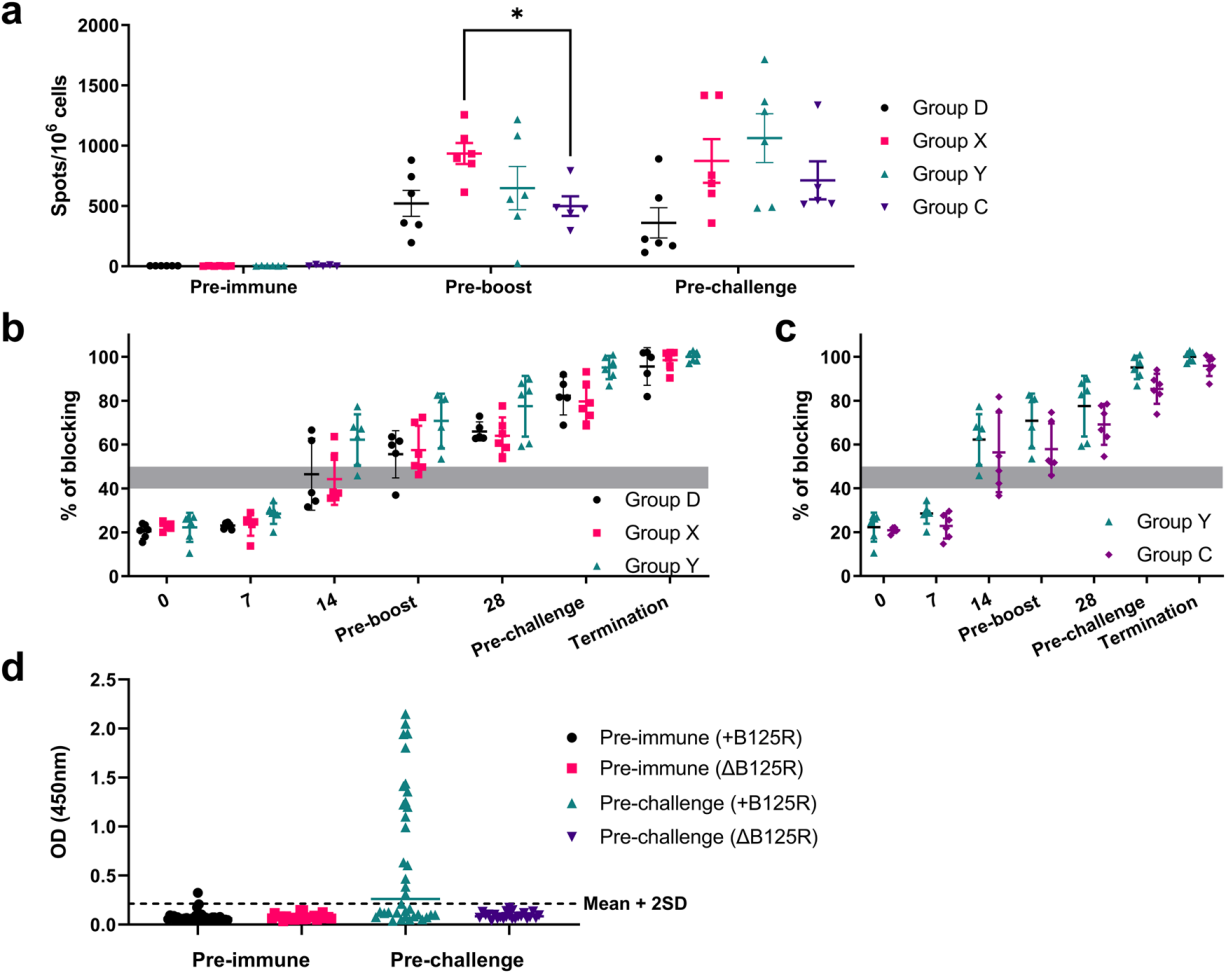
ASFV-specific immune responses following immunisation of pigs with GeorgiaΔBDKE-CmutQ96R/K108D. **a** The numbers of IFN-γ producing cells in PBMCs collected pre-immunisation, pre-boost or pre-challenge (x-axis) and stimulated with Georgia 2007/1 isolate were measured by ELISpot assays. Pigs in groups D, X, and Y were immunised and boosted with 10^2^, 10^3^, and 10^4^ TCID_50_ GΔBDKE-CmutQ96R/K108D, while Group C was only primed with 10^4^ TCID_50_ GΔBDKE-CmutQ96R/K108D. Each dot/symbol represents the mean frequencies of IFN-γ producing cells per million PBMCs from an individual pig in each group. The standard error mean (SEM) shown is for the average mean frequencies per group. Two-way repeated measure ANOVA with Tukey’s multiple comparison test was performed to find differences between the different groups, where * denotes *p* < 0.05. **b** Antibody responses of pigs were measured on different days pre and post-immunisation and challenge using a blocking ELISA against ASFV VP72 protein. Results are presented as percentage of blocking where values above 50% blocking were considered as positive antibody responses, while anything below 40% was considered as negative. Samples with blocking between 40-50% were considered as doubtful. **c** The percentage of blocking for VP72 was also compared between Group Y and Group C, where both groups received 10^4^ TCID_50_ GΔBDKE-CmutQ96R/K108D, but only Group Y was given a booster dose at 21 dpi. **d** The levels of B125R antibodies in pigs vaccinated with MLV candidates^14^ that have an intact *B125R* gene and the current vaccine MLV which has the *B125R* gene deleted (GΔBDKE-CmutQ96R/K108D) were evaluated using an in house ELISA. The cut-off of 0.214 was determined using the mean + 2 SD (standard deviation) of the 60 pre-immune serum evaluated.

The ASFV GΔBDKE-CmutQ96R/K108D vaccine candidate triggered a strong cellular response in the immunised pigs regardless of dose, as measured by the number of IFN-γ secreting cells when peripheral blood mononuclear cells (PBMCs) were stimulated with Georgia 2007/1 (Fig. 10a). When measured at 21 dpi, Group D pigs had 296.3 to 792.5 spot forming cells (SFC) per million PBMC (SFC/10^6^ cells), Group X had 615.0 to 1257.5 SFC/10^6^ cells, Group Y had 25.0 to 1217.5 SFC/10^6^ cells and Group C had 196.3 to 881.3 SFC/10^6^ cells. After the booster dose, and before challenge, four of the pigs in Group D had increased amounts of SFC/10^6^ cells, ranging from 520 to 1336.3 SFC/10^6^ cells. Pig D1 had a slight decrease to 531.8 SFC/10^6^ cells. In Group X, three of the pigs had decreased number of IFN-γ producing cells, while the other three pigs had increased number of cells. All pigs in Group Y, had increased numbers of the IFN-γ secreting cells, ranging from 483.8–1716.3 SFC/10^6^ cells. In contrast, in Group C pigs that received the same immunising dose as Group Y, but with no booster, five pigs showed decreasing amounts of IFN-γ secreting cells (115.0 to 566.3 SFC/10^6^ cells), and one pig had relatively the same number of cells as that measured before the boost (891.3 SFC/10^6^ cells). Thus, the boost appeared to have effectively increased the cellular response to ASFV in Group Y. However, the Group C pigs which did not receive a boost dose were also protected against challenge.

Anti-p72 antibodies were measured using a commercial blocking ELISA and samples above 50% blocking were considered as positive. Two pigs in Group D (D1, D5), 2 pigs in Group X (X1, X6) and 5 pigs in Group Y (Y1, Y2, Y3, Y4, and Y6) were positive at 14 dpi. Regardless of the dose, all the pigs eventually developed p72 antibodies by 28 dpi (Fig. 10b) and levels reached a saturation plateau. Immunisation regime, whether with just prime or prime and boost, did not seem to have an effect on the number of the pigs with anti-p72 antibody levels above the cut-off. All the pigs of Group C, which were not boosted, developed anti-p72 antibodies above the cut-off by 28 dpi and reached saturation (Fig. 10c).

### Low levels of shedding observed of the vaccine virus ASFV GΔBDKE-CmutQ96R/K108D

To measure shedding of GΔBDKE-CmutQ96R/K108D in oral, nasal and rectal swabs, in Experiment 2, a group of six pigs (Group E) was immunised with 10^4.5^ TCID_50_, of GΔBDKE-CmutQ96R/K108D in 1 mL of L2-RPMI (Fig. 4). This dose was selected based on 10^3.0^ TCID_50_/mL as the efficacious dose, allowing us to also evaluate its safety at 30× doses.

Three pigs of Group E (E1, E5, and E6) had temperatures above 40.5 °C. Pigs E6 and E1 had single days of raised temperatures, at 8 and 11 dpi, respectively (Fig. 5g). Pig E5 had 3 days of temperatures above 40.5 °C from 7–9 dpi. On 10 dpi, its temperature dropped to 40.1 °C, and it was lethargic, but by 11 dpi, pig E5 showed no clinical signs. (Figs. 5g and 6g). The other five pigs did not display any other clinical signs (Fig. 6g).

All six pigs were culled at 23 dpi and mild pathological lesions were observed during post-mortem examinations (Fig. 7b). Two pigs, E2 and E4, had enlargement of the tracheobronchial and renal lymph nodes, respectively. Mild presence of hydropericardium was detected in four pigs, E2, E3, E4, and E5. In pig E4 the hydropericardium showed signs of clotting and traces of fibrinous adhesions to the heart. Pig E2 had mildly non-collapsed lungs with patches of haemorrhages across the lung parenchyma. Patches of haemorrhages on the lungs were also found in pigs E3 and E6. Pig E6 also had mild ascites. No macroscopic lesions were detected in pig E1.

Viral genome was not detected in blood from two of the pigs in Group E (E3 and E4) (Fig. 8g). Mild levels of viremia, as measured by presence of viral genome copies, were detected in the blood of four pigs in Group E (Fig. 8g). Despite detecting ASFV DNA in blood from pig E1, no infectious virus was isolated (Fig. 9g). The maximum genome copies per mL in blood detected ranged from 10^3.2–3.8^, whereas the level of infectious virus ranged from 10^0.8–2.8^ TCID_50_/mL, either at 7 or 14 dpi. By 23 dpi, when these pigs were culled, neither infectious virus nor viral DNA was detected.

**Fig. 11:**
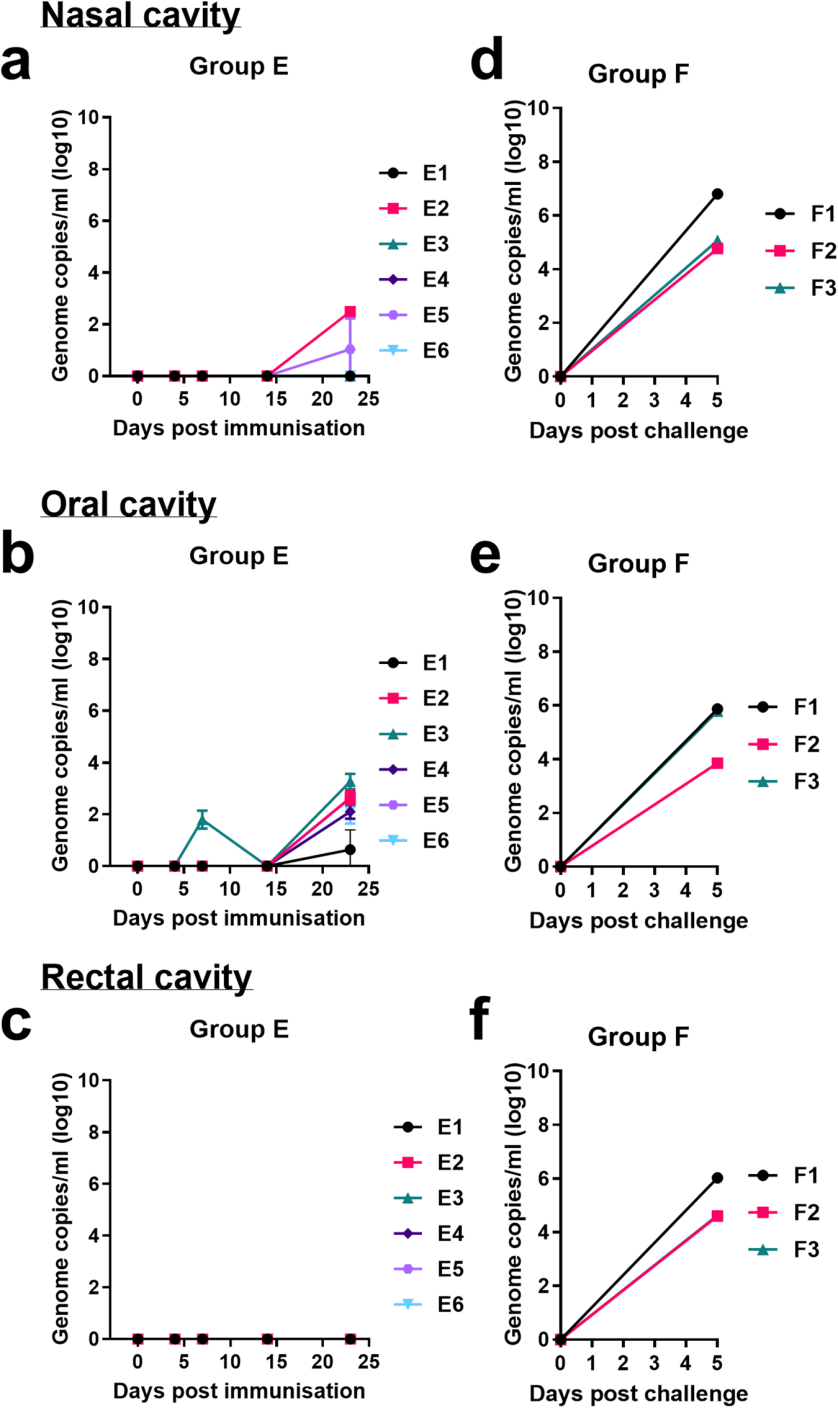
Shedding of ASFV in swabs from pigs. Viral DNA was extracted from swabs from **a** nasal, **b** oral and, **c** rectal cavities of pigs in Group E on different days until 23 dpi. These pigs were immunised with 10^4.5^ TCID_50_ GΔBDKE-CmutQ96R/K108D. As a comparison, **d** nasal, **e** oral, and **f** rectal swabs show results from swabs collected from the non-immune, challenge control pigs (Group F) from experiment 2. Genome copies per/ml log 10 are shown on the y-axis. The x-axis shows days post-immunisation or days post-challenge when samples were collected.

To evaluate the potential of vaccine virus shedding, nasal, oral and rectal swabs were collected on the same days as blood collection. Viral DNA was extracted from the swabs and the presence of ASFV viral DNA was detected by qPCR. Shedding of virus DNA in nasal swabs was detected in two pigs, one at levels below the accurate detection limit (E5) and the other at 10^2.5^ genome copies/mL PBS (E2), 23 dpi (Fig. 11a). Low levels of ASFV DNA were also detected in oral swabs from pig E3 at 7 dpi (Fig. 11b). At 23 dpi, low (10^0.6–3.3^ genome copies/mL PBS) levels of ASFV DNA were detected in oral swabs from all six pigs. No ASFV DNA was detected in the rectal cavity of the immunised pigs up to 23 dpi (Fig. 11c). Infectious virus was not detected in samples from any of the swabs from pigs in Group E.

As a comparison, pigs in the naïve control Group F were swabbed at 5 dpc when they were culled at the moderate severity endpoint. In general, moderate to high levels of ASFV DNA were detected in nasal (10^4.8–6.8^ genome copies/mL) (Fig. 11d), oral (10^3.9–5.9^ genome copies/mL) (Fig. 11e), and rectal (10^4.6–6.0^ genome copies/mL) (Fig. 11f) swabs from the non-immune pigs challenged with Georgia 2007/1. Attempts to isolate infectious virus from the swabs failed, except for a rectal swab from pig F1. However, the levels of infectious virus detected were below that required for accurate titration (data not shown).

### Antibodies against B125R protein are detected in a proportion of sera from pigs immunised with attenuated ASFV in previous experiments

Our data from screening transiently expressed B125R protein by IF for recognition by sera from pigs that were immunised with attenuated ASFV with an intact *B125R* gene, identified this protein as immunogenic. We next evaluated the numbers of the sera from the immunised pigs that recognised recombinant the B125R protein in an ELISA assay. Thirty-six pre-immune and pre-challenge serum from the pigs immunised with the attenuated ASFV vaccine candidates (GΔKE-CmutQ96R, GΔKE-CmutQ96R/K108D, and GΔDKE-CmutQ96R/K108D) that still have intact *B125R* genes from our previous study^14^ and twenty-three from the current study (GΔBDKE-CmutQ96R/K108D) were used to evaluate the antibody response to B125R protein. Previously, using an immunofluorescence assay, to detect transiently expressed B125R, 75% of sera tested were positive (See section Results: *Identification of B125R as an immunogenic protein)*. However, using an in-house ELISA with recombinant B125R protein only 52.8% of the pre-challenge sera tested positive (Fig. 9d). All the pre-challenge sera from the current study were negative for anti-B125R antibodies. However, one of the pre-immune samples from the screen for recognition of transiently expressed B125R gave values above the cut-off, giving the B125R ELISA a 98.8% specificity (Fig. 9d). For this B125R ELISA, the cut-off value, based on pre-immune sera, was at least 2-fold higher than the values obtained when the same sera were tested using an in house ASFV CP204L/P30 ELISA (data not shown). Thus, improving the ELISA by using a different protein purification system and/or an alternative assay for detection of antibody may increase the number of positive samples detected. This would support the use of the B125R protein as a negative DIVA candidate in combination with other proteins. Using additional proteins encoded by other deleted genes, for example *EP153R*, may also improve a DIVA test for this vaccine candidate.

## Discussion

Vaccines are not widely available for ASFV although two gene-deleted modified live ASFV vaccines have recently been approved for limited field use in Vietnam. One of these has a single deletion of the *I177L* gene, ASFV-G-ΔI177L^11,26–28^ and the other, ASFV-G-ΔMGF, has six members of multigene families (*MGF 360 or MGF 505* deleted)^13^. Both viruses were shown to induce high levels of protection across a range of immunising doses. In a safety trial for reversion to virulence during passage in pigs, genome rearrangements of the ASFV-G-ΔMGF virus were observed and a partial reversion to virulence occurred although pigs recovered by the end of the study^29^. In another study the same six *MGF 360 and 505* genes were deleted from a Chinese ASFV isolate. Although this virus was also attenuated and induced high levels of protection, reversion to virulence was demonstrated in a pig passage study. An additional gene, *EP402R*, which codes for the CD2v protein, was deleted. This virus, HLJ/18-7GD, also conferred high levels of protection against challenge. Safety was improved since reversion to virulence was not observed in the pig passage study^12^. Further gene modifications including deletion of *MGF110-5L and -6L* occurred to the single gene deleted *I177L* virus following passage in a cell line. This modified virus also retained the ability to induce good levels of protection in pigs^30^.

Although these MLV in use in Vietnam have shown good potential to be used more extensively in the field, further testing is required. Further knowledge about the persistence of the vaccine viruses in the field, either by horizontal transmission between vaccinated and naïve pigs in contact or by persistence of virus in tissues of individual animals, would be valuable. Higher levels of replication and persistence of virus in blood or tissues could also increase the chances of recombination between virulent isolates circulating in the field and the MLVs. A complex genotype I/genotype II recombinant virulent ASFV, with multiple cross-over points, has been isolated from several locations in the field in China and Vietnam^6,31^. It is presumed that the virulent recombinant genotype II/I virus was derived by recombination between a high virulence GII virus and the low virulence GI virus. Regions in ASFV genomes which may more frequently undergo recombination include the MGF genes and the *EP402R* gene^32^. However, evidence is lacking for recombination between other viruses circulating in the field.

Many non-essential genes ASFV genes have been identified which when deleted reduce virus virulence and induce protection. However, identifying optimal combinations of gene deletions to obtain acceptable levels of safety and efficacy is difficult. Several other promising candidate MLVs have been tested by immunisation and challenge of pigs in small-scale experiments^14,33–36^ but data are not yet available on field trials conducted.

Previously, we developed a protective multiple gene-deleted ASFV GII expressing a non-HAD version of the CD2v protein^14^. Our aim was to develop a candidate vaccine, which replicated well in macrophage cultures, induced mild or no clinical signs, low and transient viremia in pigs, and high levels of protection. We showed that this multi-gene deleted genotype II virus, GΔDKE-CmutQ96R/K108D, induced dose-dependent protection of 83.3 to 100% against challenge with the virulent genotype II ASFV. However, in subsequent experiments one pig was culled at the moderate severity humane endpoint following immunisation with 5 × 10^4^ TCID_50_. To improve the safety of GΔDKE-CmutQ96R/K108D, we decided to delete another gene. We chose to delete *B125R* because an initial screen of transiently expressed ASFV proteins for recognition by the sera from immunised pigs, identified B125R as a potential negative serology marker for DIVA. To improve the safety and evaluate the use of B125R as a negative serological DIVA marker, we deleted the *B125R* gene from GΔDKE-CmutQ96R/K108D. Immunisation and challenge experiments in pigs showed that the protective efficacy of this *B125R* gene deleted virus, GΔBDKE-CmutQ96R/K108D, was higher than the original virus GΔDKE-CmutQ96R/K108D, since 100% protection was observed across doses from 10^2^ to 10^4^ TCID_50_ (a summary of these results is provided in Supplementary Table 2). One pig immunised with the lowest dose, 10^2^ TCID_50_, reached the defined moderate severity humane endpoint and was culled at 7 dpi. Moderate levels of viremia and moderate lesions typical of ASF disease were observed in this pig suggesting it may have recovered. These results suggested that the process of deleting the *B125R* gene may have reduced rather than increased attenuation of the virus. One possibility is that the *B125R* gene may have a role in reducing ASFV pathogenesis so that deleting the gene results in increasing replication in vivo and pathogenesis. However, this seems unlikely since deleting the *B125R* gene from a virulent Chinese isolate, SY18, resulted in a slight virus attenuation. This may have resulted from an accompanying frame shift mutation in the *A104R* gene rather than deletion of the B125R gene^37^. The *A104R* gene has previously been shown to be non-essential and deletion resulted in dramatic attenuation of virulent virus^38^. An alternative explanation to explain reduced attenuation and accompanying increased efficacy of the *B125R* gene deleted virus, GΔBDKE-CmutQ96R/K108D, may be due to the removal of the fluorescent protein marker gene cassettes. The p30 promoter controls expression of both fluorescent protein genes used in the parental GΔDKE-CmutQ96R/K108D virus. TTS was not included downstream of the reporter genes in this virus. Read through transcription from the p30 promoter, which is active at both early and late times of infection, is likely to have extended into downstream genes *K421R* or *EP364R*. Transcription read through is known to occur during ASFV replication^20,39^. Evidence is that the first open reading frame downstream from the transcription initiation site is the predominant translated protein. Transcription initiation of mRNAs from the gene-specific promoters for *K421R* and *EP364R* may be reduced if significant read-through transcription occurred from the upstream genes. This would result in reduced translation of these protein products. In the case of the recombinant viruses, transcription controlled by the p30 promoter upstream of the fluorescent protein reporter genes, may read into downstream ORFs. In the GΔBDKE-CmutQ96R/K108D virus TTS were included downstream of the reporter cassette to avoid potential transcription interference of downstream genes (Fig. 2b).

Low or no viremia was detected after challenge except for 1 pig in each of Groups X and Y, which had moderate levels of replication. A comparison between single or prime and boost vaccination regimes before challenge of the pigs indicated that the boost increased the numbers of by IFN-γ secreting cells measured by ELIspot assay. However, 100% protection was achieved in both groups. Possibly the prime and boost immunisation regime may increase the duration of immunity, but this remains to be determined. In summary, we have improved the efficacy of our previous parental gene deleted virus GΔDKE-CmutQ96R/K108D by further modifications of the genome including the removal of the genes coding for fluorescent proteins followed by deletion of the *B125R* gene. As this recombinant, GΔDKE-CmutQ96R/K108D, carries only one red fluorescent marker, further modifications could be made by using a green fluorescent protein gene as marker. The intermediate virus lacking fluorescent reporter genes that we generated could also be used as a template to delete one or two additional genes. Deleting additional genes to increase safety but maintain protection could therefore be achieved. Further testing of these experimental MLVs would be required before they could be considered to take further toward licensing. Removal of exogenous DNA sequences from the MLV, including reporter gene cassettes, may also be required. We identified that the B125R protein could contribute to a DIVA test to differentiate infected from vaccinated pigs, although additional negative serology markers would be required for field use. Further testing, including comparing sera collected pre-immunisation, pre-challenge and after challenge would also be required before eventual use of a DIVA test in the field. The cross-protective potential of GΔBDKE-CmutQ96R/K108D against other genotypes including genotype I and genotype II genotype I hybrids could also be tested in future.

## Materials and methods

### Cells

Vero cells were maintained in DMEM supplemented with 10% foetal bovine serum (FBS) and 1% penicillin–streptomycin (Pen–Strep). Porcine bone marrow cells (PBMs) were obtained from the leg bones of 4- to 5-week-old outbred Landrace-Large White pigs and were cultured in EBSS supplemented with 10% pig serum, 1% Pen-Strep and 1% HEPES. PBMs were further purified using gradient-centrifugation on Histopaque-1083, at 1.083 g/mL density. Purified PBMs were cultured in RPMI supplemented with 10% FBS, 1% Pen–Strep and 100 ng/mL porcine CSF1^40^. The MLV virus used in pig immunisation experiments was passaged twice in a proprietary cell line and provided ready-to-use by Zoetis. Adaptation of the MLV virus to the cell line was not required and virus titres obtained from the cell line were similar to those in PBM-derived cells and did not alter during passage.

### Viruses

The ASFV Georgia 2007/1 and the non-HAD recombinant derivative, GeorgiaΔDP148RΔK145R∆EP153R-CD2v_mutantQ96R/K108D (here named GΔDKE-CmutQ96R/K108D) have been described previously^14^. Virus titrations were carried out in PBM-derived cells in quadruplicates by haemadsorption assay (HAD_50_/mL) or by end-point titres based on the expression of reporter protein, β-GUS or fluorescent reporter protein, tdTomato (TCID_50_/mL). Titres were calculated using the Spearman and Kärber algorithm^41^. Titrations of viruses for use in immunisation were measured in cells from three separate pigs each time by three different people.

### In vitro screen to detect antibodies against B125R in sera from pigs infected with ASFV

Vero cells on coverslips were transfected with a plasmid (pcDNA3.1) harbouring the *B125R* gene under control of the human cytomegalovirus immediate early promoter using TransIT LT-1 (Mirus Bio). The *B125R* open reading frame was fused with an HA epitope tag at its 3′ end. After 48 h, the transfected cells were fixed with 4% paraformaldehyde, permeabilised with 0.2% Triton X-100 and blocked with 1% bovine serum albumin in 0.1% PBS-Tween20 (PBS-T). To evaluate the potential of B125R as a DIVA target, pre-immunisation and pre-challenge sera from previous animal experiments were used as primary antibody^15–17^. In parallel a rat anti-HA antibody was used to detect the cells transfected with plasmid expressing B125R. After incubation and washes, secondary antibodies, goat anti-porcine Alexa Fluor 488 (Southern Biotech) or goat anti-rat Alexa Fluor 568 (Invitrogen), were added and further incubated. The cells were then washed before counterstaining with DAPI (Sigma) to detect nuclei. Coverslips were mounted in Vectashield antifade mounting medium, sealed and imaged under the confocal microscope, Leica SP8 CLSM.

### Generation of a derivative of GΔDKE-CmutQ96R/K108D lacking the B125R gene

To delete *B125R* gene from parental ASFV GΔDKE-CmutQ96R/K108D, a 2-step sequential protocol was undertaken^18^. This was necessary since this virus already contains gene cassettes for expression of 2 fluorescent reporter proteins (TagRFP-T and mNeonGreen) and a gene for β-GUS reporter protein. No additional fluorescent protein marker genes were available for isolation of new recombinant viruses and the reporter cassettes were therefore first removed.

### Removal of fluorescent protein marker genes from GΔDKE-CmutQ96R/K108D

The non-HAD, GΔDKE-CmutQ96R/K108D virus was constructed with the reporter cassettes flanked on both sides by loxP sites in the same orientations (Fig. 2a). This enabled removal of those cassettes by recombination mediated by the Cre recombinase^18^.

Purified PBMs were first infected with GΔDKE-CmutQ96R/K108D at a multiplicity of infection (MOI) of 0.2. After 3 h incubation at 37 °C, the cells were transfected with pEX-K4_p72_Cre recombinase, a plasmid expressing Cre recombinase under control of the ASFV *B646L/p72* promoter^18^. Forty-eight hours later, the TagRFP-T^neg^mNeonGreen^neg^ cells, negative for expression of both fluorescent proteins, were bulk sorted in 1 mL of complete RPMI with a FACSAria fusion cell sorter (BD Biosciences). The sorted TagRFP-T^neg^mNeonGreen^neg^ cells were postulated to contain both the uninfected cells and the infected cells from which both TagRFP-T and mNeonGreen genes have been deleted by the Cre recombinase. Limiting dilutions were then performed on these cells and wells containing the infected cells were identified using 3 criteria: (i) no expression of fluorescent protein markers; (ii) CPEs and (iii) further confirmation of infection by qPCR for the ASFV *B646L/p72* gene^42^. The final selected non-HAD, non-fluorescent recombinant virus, GΔDKE-CmutQ96R/K108D-NoFP (Fig. 2a), was then grown in PBMs and used in the subsequent step to delete the B125R gene.

### Deletion of the ASFV *B125R* gene from GΔDKE-CmutQ96R/K108D-NoFP

A transfer plasmid, pΔB125R-tdTomato, containing: (i) a 521 bp fragment B125R left flanking region (FR682468.2, positions 106064–106584), (ii) a reporter gene, expressing the tdTomato fluorescent protein^43^, under the control of ASFV p30 promoter^44^, flanked by 2 loxP sites in the same direction^18^, and (iii) a 515 bp B125R right flanking region (FR682468.2, positions 106887–107401) was cloned (Fig. 2b). Plasmid DNA was commercially synthesised and transformed in *E. coli* (DH5α) and extracted (QIAprep Spin Mini Kit Qiagen).

Purified PBM-derived cells were infected with GeorgiaΔDKE-CmutQ96R/K108D-NoFP at 0.5 MOI and transfected with the transfer plasmid, pΔB125R-tdTomato, using TransIT-X2 (Mirus Bio). Forty-eight hours later, single cells expressing tdTomato were sorted into 96-well plates containing purified PBMs. Recombinant virus, GeorgiaΔB125RΔDP148RΔK145R∆EP153R-CD2v_mutantQ96R/K108D (abbreviated to GΔBDKE-CmutQ96R/K108D), was further purified by two rounds of single cell sorting and two rounds of limiting dilutions. DNA extracted from purified recombinant viruses was tested by PCRs and Sanger sequencing to confirm deletion of the *B125R* gene, absence of parental virus and insertion of the reporter cassette at the correct position.

### Whole genome sequencing of GΔBDKE-CmutQ96R/K108D

Viruses were grown in purified PBMs, and 4 days post-infection, the cells and supernatants were harvested. Cells were pelleted by centrifugation at 300 g for 10 m. Supernatants containing extracellular virus was treated with DNase I to degrade cellular DNA. The virus was concentrated, and viral DNA was extracted using MagAttract HMW DNA kit (Qiagen). DNA libraries were prepared using Illumina DNA prep kit (Illumina) and sequenced on a MiSeq instrument using a 600 cycle v3 cartridge. A total of 3,663,204 sequence reads were obtained and 95.2% were assembled using the NGS-based whole genome variant analysis module in SeqMan NGen (DNASTAR Lasergene 17), using ASFV Georgia 2007/1 (Accession number: FR682468.2) as reference genome. The median coverage depth was 4837.4. The sequence data was submitted to the NCBI SRA database accession number PRJNA1227411.

### Multistep growth curve

Isolated PBMs, purified on Histopaque gradients, were infected at a MOI of 0.01 in triplicates. Total infectious viruses were harvested from supernatants and cells after freeze-thawing three times. Cellular debris was discarded after centrifugation and then samples were titrated on PBMs. Repeated measure two-way analysis of variance, with Dunnett’s multiple comparisons test, was performed to compare the replication of the recombinant and wildtype ASFV strains.

### In vivo immunisation and challenge of pigs

All animal experiments were conducted in Specified Animal Pathogens Order Group 4 high-containment animal housing at the Pirbright Institute according to regulated procedures from the Animals Act UK 1998 and conducted under Home Office License 7088520. Two separate experiments were conducted to evaluate clinical signs following immunisation of pigs with GΔBDKE-CmutQ96R/K108D and protection following challenge with homologous virulent virus Georgia 2007/1.

Groups of six or three, for the control non-immunised animals, landrace-large white female pigs between 18 and 22 kg were randomly assigned to groups and settled in for 7 days before the start of the experiment. Female pigs were used to avoid behaviour issues that may occur if mixed sex animals were used. Figure 4 shows a timeline of experiments carried out, including details of the virus and dose used for immunisation and challenge by the intramuscular route. Baseline rectal temperatures were recorded for 3 days before immunisation. Clinical scores including rectal temperatures were recorded daily in the morning as described previously^45,46^. If clinical signs developed, pigs were inspected more regularly. For immunisation and collection of blood samples, pigs were restrained using a snatch while the procedure was completed. All staff handling animals had the required personal licences and their competency was reviewed regularly. Pigs were immunised intramuscularly in a volume of 1 mL in the neck behind the ears, avoiding introduction of air bubbles in the inoculum. Blood samples were collected by venipuncture of the anterior vena cava using a needle appropriate for the age of the pig. The volume collected did not exceed 10% of the estimated total volume on any one time point or 15% of the total blood volume in any 28-d period. Blood volume is calculated at 65 mL per kg mass of the animal. After bleeding, pressure is applied to halt any blood flow and animals are checked at intervals to confirm their wellbeing. Two experiments were carried out in which pigs were immunised with GeorgiaΔBDKE-CmutQ96R/K108D intramuscularly with different doses. At the moderate severity humane endpoint, defined on the Home Office License, or at termination of the experiment, pigs were restrained using a snatch and a lethal dose of anaesthetic administered in the vena cava. Dolethal (200 mg/mL) was given intravenously at a dose of 0.7 mL per kg of the pig bodyweight. Permanent cessation of circulation was confirmed. In experiment one, Group X was immunised with 10^3^ TCID_50_ and Group Y with 10^4^ TCID_50_ (Fig. 4). At 21 dpi, pigs were boosted with the same vaccine by the same route and respective doses. Pigs were challenged, alongside a naïve control group of 3 pigs (Group AA), with 10^3^ HAD_50_ Georgia 2007/1 at 42 dpi (Fig. 4). In the second experiment, 3 groups of six pigs were immunised with GeorgiaΔBDKE-CmutQ96R/K108D. In this experiment, the inoculum included L2 stabiliser in RPMI. This allowed us to also evaluate the impacts of the stabiliser on clinical signs and induction of protection. L2 stabiliser is composed of as follows: Dextran 40, Casein hydrolysate, Lactose monohydrate, Sorbitol 70% (solution), Sodium hydroxide. L2 stabiliser has been used in Suvaxyn CSF marker, a licensed vaccine without affecting protection of pigs against classical swine fever. One group was immunised and boosted intramuscularly with 10^2^ TCID_50_ (Group D) (Fig. 4). A second group of six pigs (Group C) were intramuscularly immunised with 10^4^ TCID_50_ GeorgiaΔBDKE-CmutQ96R/K108D, but these pigs were not given the booster dose. The pigs of both Groups C and D were then challenged at 44 dpi with 10^3^ HAD_50_ of Georgia 2007/1. Group F, a group of three naïve pigs was challenged in parallel. A third group of six pigs (Group E) was immunised with 10^4.5^ TCID_50_ to evaluate safety and vaccine shedding via the nasal, oral and rectal routes. These pigs were terminated at 23 dpi. Serum and whole blood in EDTA samples were collected at selected time points. The nasal, oral, and rectal swabs of the Group E pigs were collected using sterile swabs in PBS supplemented with 1% penicillin and streptomycin (1% Pen-Strep).

Pigs were culled when they reached a defined moderate severity humane endpoint, (UK Home Office project license7088520) or at the end of the experiments. Post-mortem macroscopic findings were recorded^23,46^.

### ASFV detection

DNA was extracted from whole blood in EDTA and oral, nasal and rectal swabs in PBS. DNA from the blood and swabs was extracted in duplicate using the MagMAX™ CORE Nucleic Acid Purification Kit on a Kingfisher Flex Extraction System. Extracted DNA was amplified in duplicate for viral DNA detection by the *B646L/p72* -targeting qPCR on an AriaMx real-time PCR system (Agilent) following a modified protocol^42^^,45^. Standard curves from a p72 mimic plasmid were included in every run to allow quantification^42^. Results are reported as log_10_ genome copies/mL. The cut-off for accurate detection is 10^2^ ASFV genome copies/mL; below this, the exact genome copies cannot be determined. The levels of the virus genome copies were defined as low if ≤10^4^, moderate if between 10^4^ and 10^6^; and high if ≥10^6^ genome copies per mL.

For quantification of infectious virus, samples were serially diluted and titrated in PBM-derived cells by HAD50/mL or TCID50/mL in quadruplicate.

### Anti-p72 antibody responses in the immunised pigs—ELISA

Sera collected throughout the experiments were separated by centrifugation and used in a commercial blocking ELISA (INgezim PPA Compac, Ingenasa) to measure antibody responses against the ASFV p72. The final optical density (OD) was read at 450 nm on a BioTek microplate reader. The percentage of blocking was calculated using the following formula: [(negative-control OD − sample OD)/(negative-control OD − positive-control OD)] × 100. Samples >50% blocking were considered as positive, while anything <40% was considered as negative. Samples with blocking between 40 and 50% were considered as doubtful.

### Anti-B125R antibody responses in the immunised pigs—ELISA

B125R antigen, expressed in HEK293T cells as a secreted form with His tag at the C-terminus to aid purification, was coated at 2 µg/mL (diluted in PBS) on MaxiSorp™ plates (Nunc) overnight at 4 °C. Coated plates were incubated with blocking buffer (5% milk in PBS-T,) for 1 h at 37 °C. Pre-immunisation and pre-challenge sera from the pig immunisation and challenge experiments described above and from previous experiments^14^ were diluted at 1:200 in blocking buffer. Diluted sera were added onto plates and incubated at 37 °C for another hour. Plates were then washed with PBS-T and further incubated with 1:20,000 diluted goat anti-pig IgG (H + L) antibody, HRP conjugated (Bethyl Laboratories), at 37 °C for 1 h. Bound antibodies were detected by treatment with 1:1 tetramethylbenzidine substrate and hydrogen peroxide at room temperature for 10 to 15 min. The reaction was stopped with 2 M H_2_SO_4_ and absorbances were measured at 450 nm on a BioTek plate reader with Gen5 software. The cut-off was determined using pre-immune sera from the 60 pigs: Mean + 2 SD, the cut-off value in this experiment was 0.214.

### IFN-γ secreting cells in pigs—ELISpot assay

PBMCs from the blood of pigs collected before immunisation, boost and challenge were purified by centrifugation on Histopaque®-1077 Hybri-Max gradients. The levels of IFN-γ producing cells were measured using an ELISpot assay^14^ and the results are presented as SFC per million PBMCs.

## Supplementary information

Supplementary data

## Data Availability

Sequence data that support the findings have been deposited in the NCBI SRA database with the primary accession code PRJNA1227411. Other data are provided in the manuscript or supplementary information files.
