# Supplementary material for: Deletion of B125R increases protection induced by a genotype II African swine fever vaccine candidate
Source: NPJ Vaccines. 2025 Mar 19;10:52. doi: 10.1038/s41541-025-01101-4 (PMC11923233; doi:10.1038/s41541-025-01101-4)
Supplement: Supplementary file 1 — Supplementary data [file 41541_2025_1101_MOESM1_ESM.pdf]

Supplementary data

Deletion of B125R increases protection induced by a genotype II African swine fever vaccine candidate

Anusyah Rathakrishnan[1], Ana Luisa Reis[1], Katy Moffat[1], Lynnette Goatley[1], Elisenda Viaplana[2], Jose Carlos Mancera[3], Alicia Urniza[2], Linda K. Dixon[1*]

Supplementary Figure

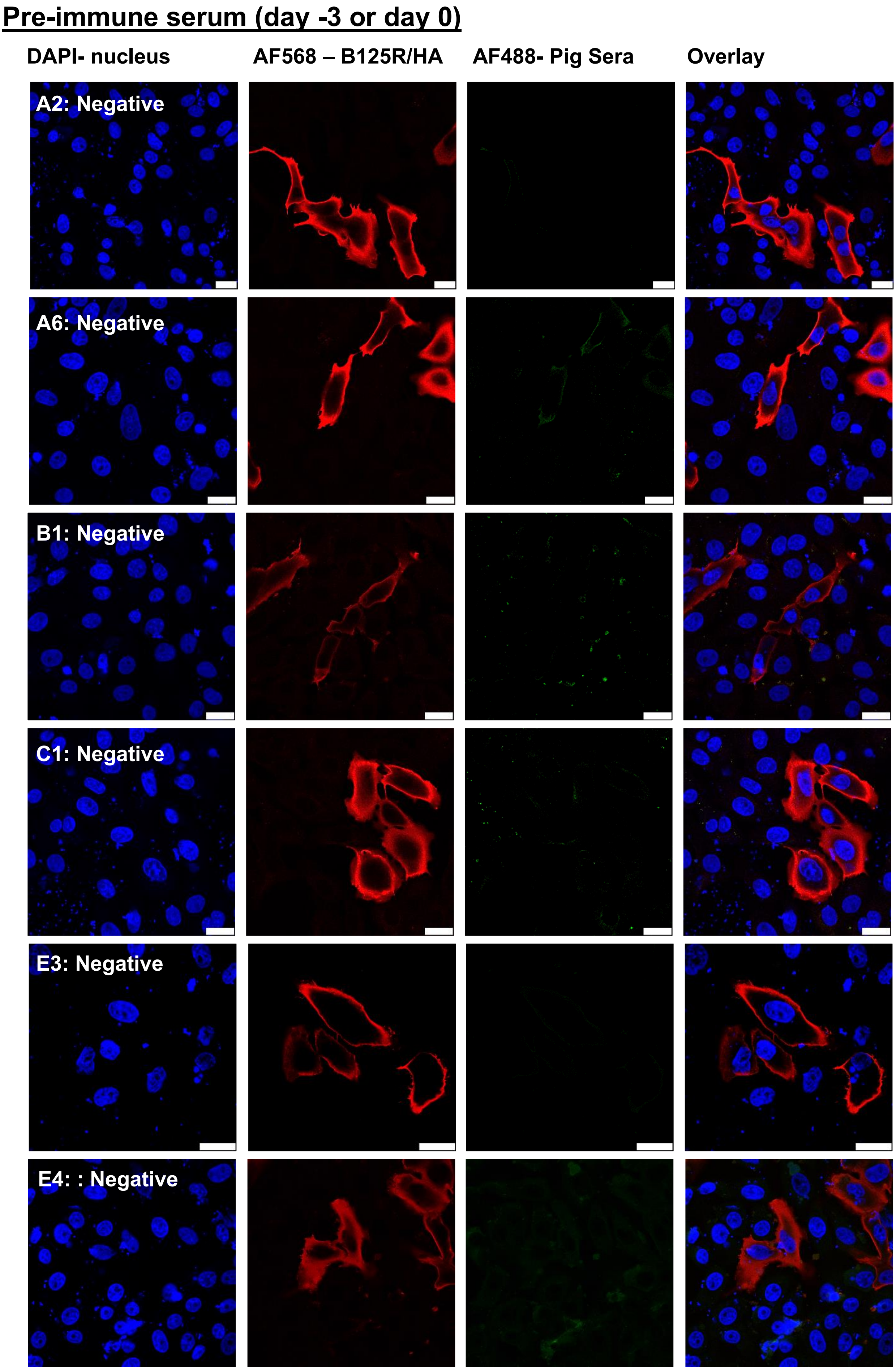

GeorgiaΔMGF immunized pigs (34 dpi)

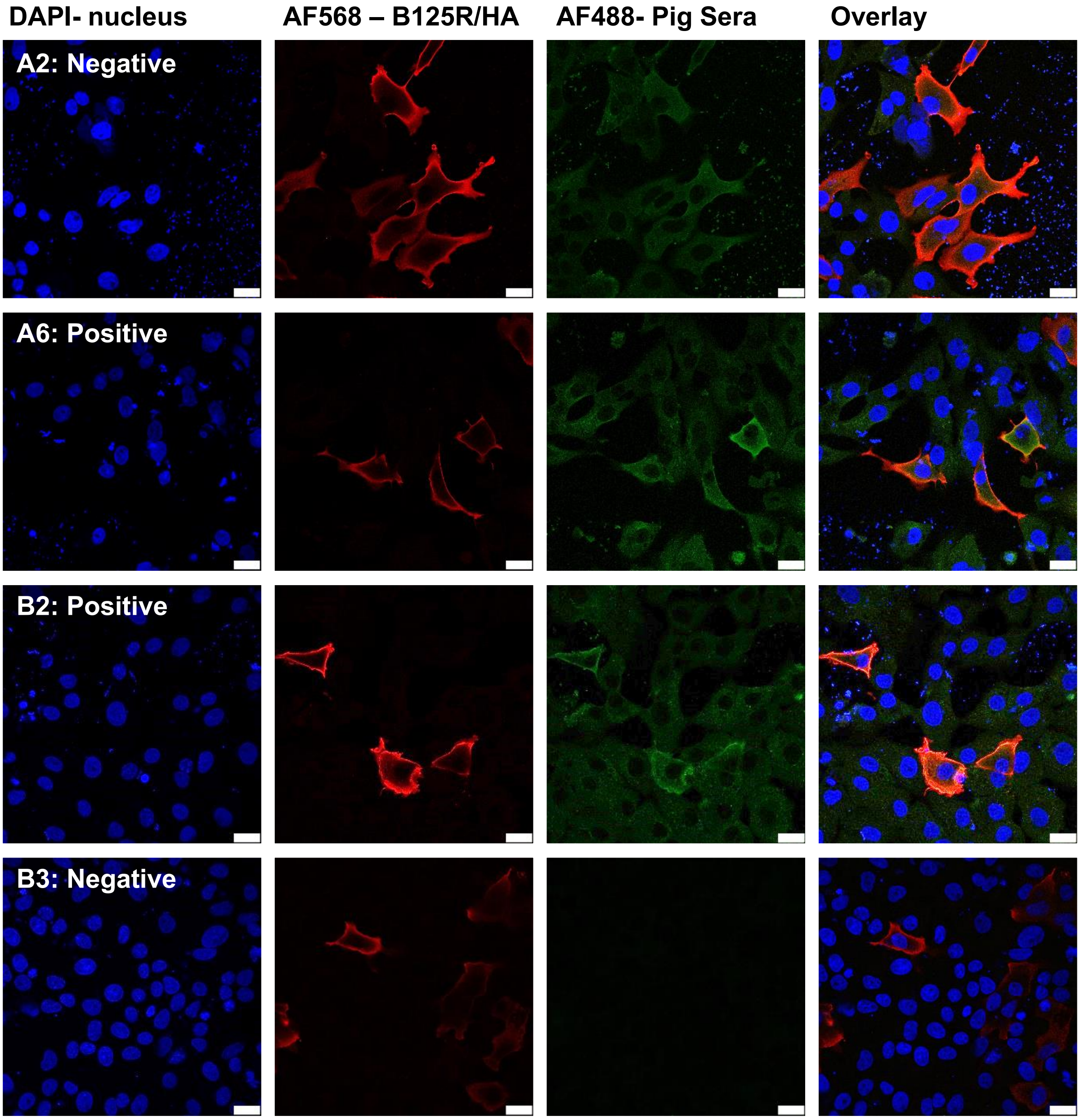

BeninΔMGF immunized pigs (38 dpi)

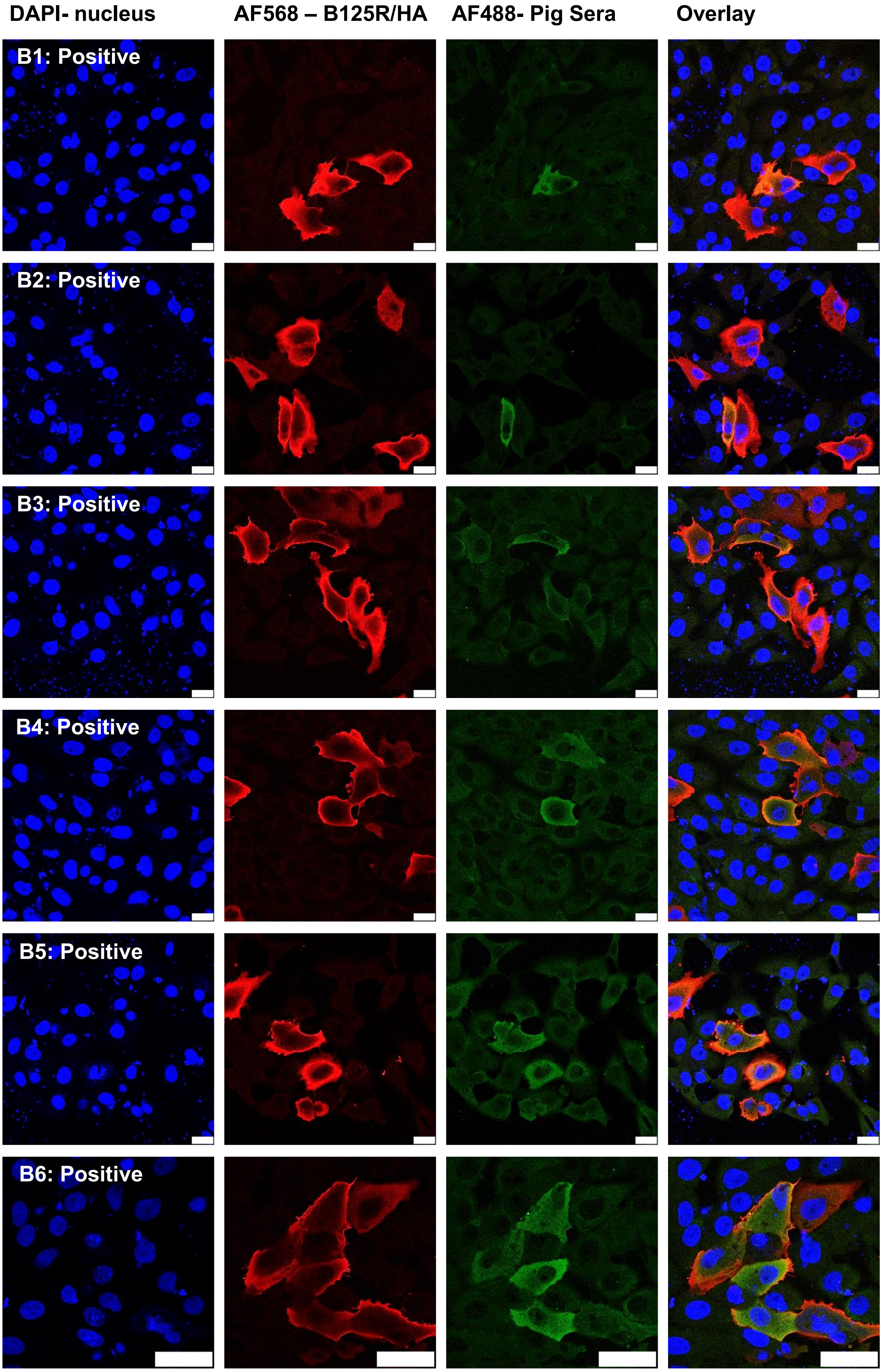

OURT88/3 immunized pigs (20 dpi)

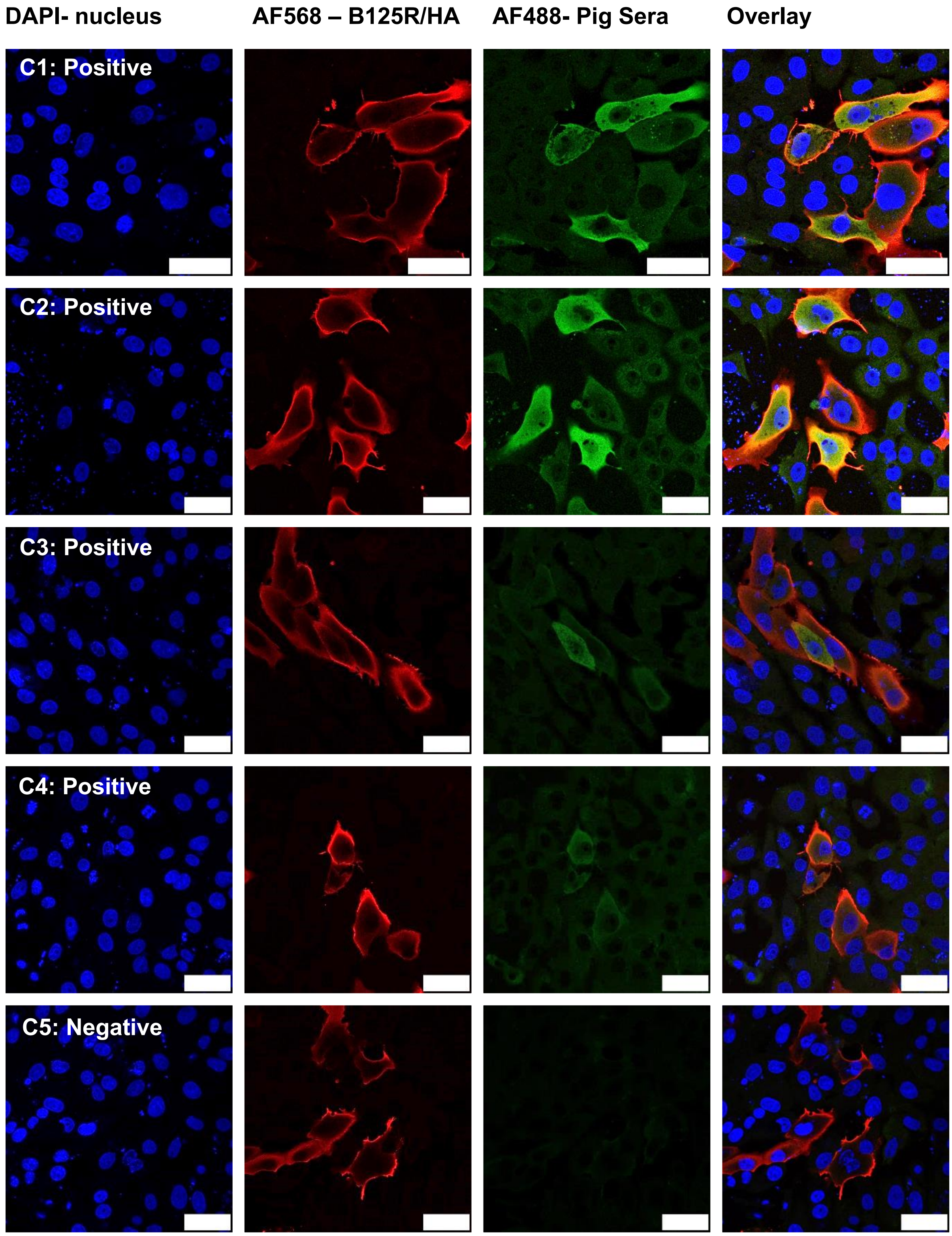

BeninΔDP148R immunized pigs (38 dpi)

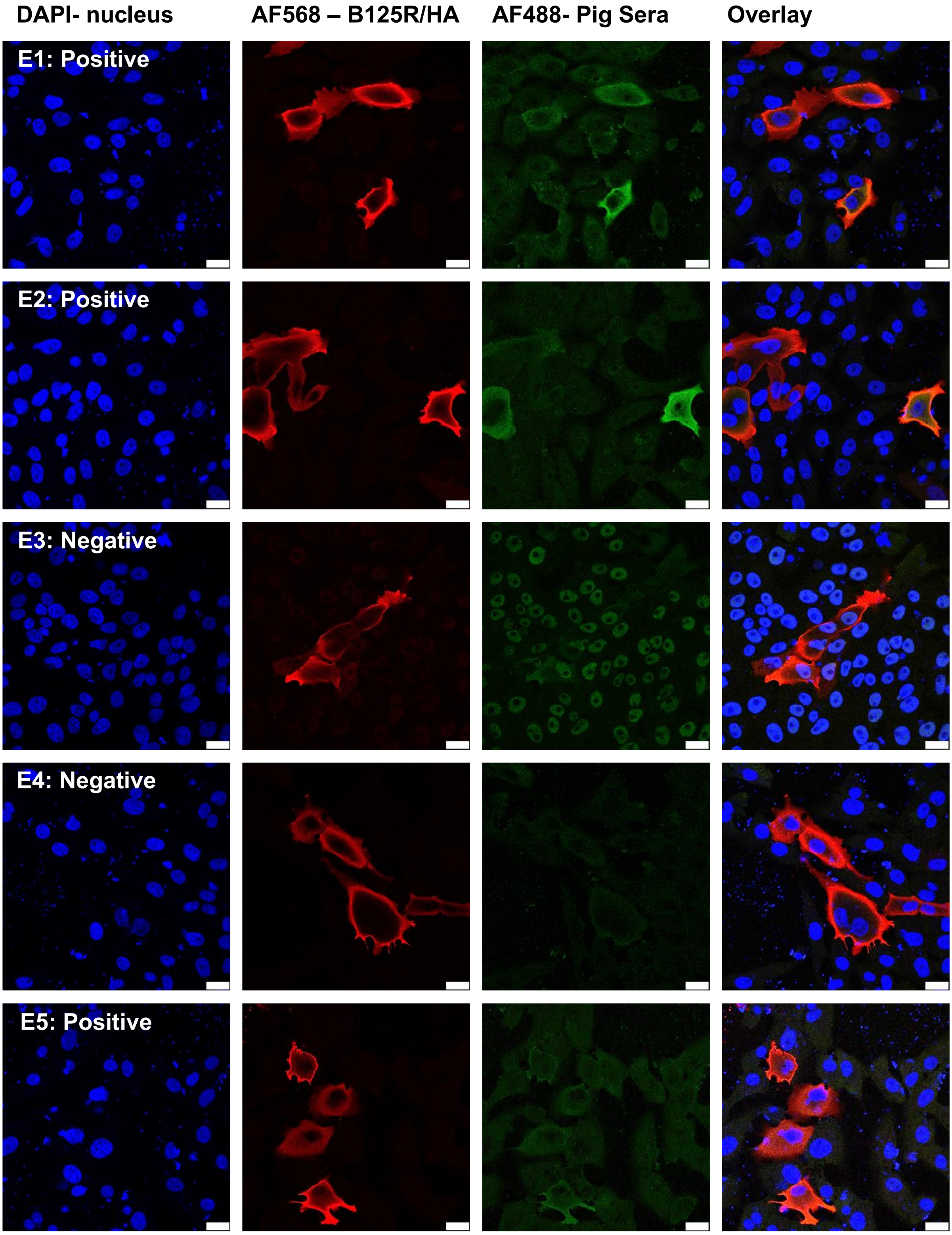

Supplementary Figure 1. Screening for B125R antibodies in sera from immunised pigs. A plasmid expressing ASFV Georgia B125R protein was transfected into Vero cells. After 48h, the cells were fixed. Cells on coverslips were permeabilized and probed with rat anti-HA, to detect the C-terminal HA tag on the B125R protein and with the pre-immune and pre-challenge pig sera from previous vaccination experiments. These included experiments in which pigs were immunised with BeninΔDP148R, GeorgiaΔMGF, BeninΔMGF or OURT88/3 as indicated. Appropriate secondary antibodies were incubated to detect bound antibodies including goat anti-rat Alexa-Fluor 568 and goat anti-pig Alexa-Fluor 488, and were counterstained with DAPI to show the nucleus (blue). Individual panels show the expression of HA-tagged B125R (red), B125R detected by antibodies in pig sera (green) and DAPI (blue). The last column shows the overlay from the 3 different channels. The scale bar represents 25µm. The colours have been brightened using PowerPoint to aid visualisation.

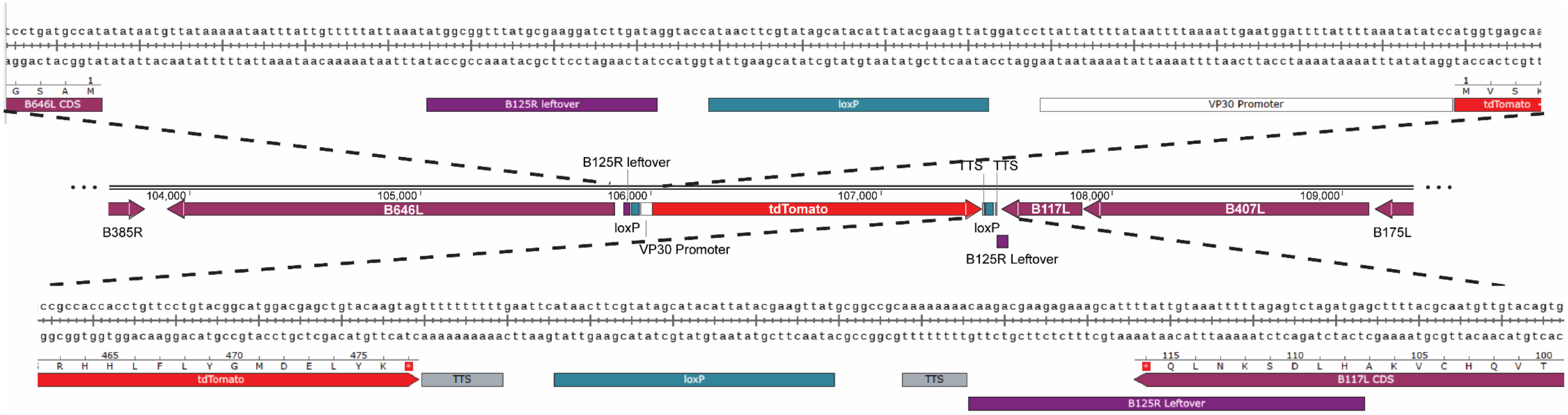

Supplementary Figure 2. GeorgiaΔBDKE-CmutQ96R/K108D sequences at site of recombination. Sequence of tdTomato fluorescent protein reporter cassette and ASFV genome regions flanking the B125R gene deletion in recombinant virus GΔBDKE-CmutQ96R/K108D. To confirm the expected deletion of the *B125R* gene and insertion of the *tdTomato* reporter cassette both Sanger sequencing of a PCR product amplified across the insertion site from genomic DNA and whole genome sequencing of GΔBDKE-CmutQ96R/K108D were carried out. The genome map of this region is shown in the centre of the figure. Above this is shown the sequence and map of the genome region at the 5' end of the *tdTomato* reporter gene and below the genome region at the 3' end. The positions are indicated of different elements including flanking ASFV genome regions, inserted ASFV Transcription Termination Sites (TTS), lox P sites, ASFV p30 promoter and *tdTomato* gene sequences. Figure 2 shows how the GΔBDKE-CmutQ96R/K108D recombinant virus was derived and this is explained in the Results section. Numbering indicates positions on the recombinant ASFV GΔBDKE-CmutQ96R/K108D genome.

Supplementary Tables

Supplementary Table 1: Changes in GeorgiaΔBDKE-CmutQ96R/K108D compared to the parental MLV, GeorgiaΔDKE-CmutQ96R/K108D and wildtype Georgia 2007/1. These modifications were confirmed by whole genome sequencing.

	**Georgia (FR682468.2)**	**GΔDKE-CmutQ96R/K108D**	**GΔBDKE-CmutQ96R/K108D**
**K145R**	65719 to 66156	Deletion of K145R from 65719 - 66071. Substituted by TagRFP-T	K145R remains deleted. TagRFP-T is deleted, leaving behind a single LoxP.
**EP153R**	73808 to 74284	Deletion of EP153R. Substituted by mNeonGreen.	EP153R remains deleted. mNeonGreen is deleted, leaving behind a single LoxP.
**EP402R**	74354 to 75436	Q at a.a. position 96 mutated to R. K at a.a. position 108 mutated to D.	Q96R and K108D substitutions remain, without additional changes.
**B125R**	106557 to 106934	B125R is intact	B125R deleted from 106584 - 106886. Substituted by tdTomato.
**DP148R**	184332 to 185045	Deletion of DP148R from 184193 to 185020. Substituted by β-GUS	DP148R remained deleted. GUS remained intact.

Supplementary Table 2: Summary of GeorgiaΔBDKE-CmutQ96R/K108D as a modified live vaccine candidate for ASF.

			Pre-challenge						Post-challenge						
			Clinical signs		ASFV DNA[g]		Viremia[c,g]			Clinical signs		ASFV DNA[g]		Viremia[d, g]	
	Prime[f]	Boost[f]	>40.5°C	Signs[a]	# pigs	GC/mL[b]	# pigs	$TCID_{50}$/mL	Protection	>40.5°C	Signs[a]	# pigs	GC/mL[b]	# pigs	HAD_{50}/mL
**Group D**	$10^{2.0}$	$10^{2.0}$	3/6	1/6	3/6	$10^{1.9 - 6.4}$	2/6	$10^{0.8 - 5.3}$	100%	2/5	2/5	2/5	$10^{0.4 - 5.5}$	2/5	$10^{1.0 - 5.5}$
**Group X**	$10^{3.0}$	$10^{3.0}$	1/6	0/6	4/6	$10^{2.0 - 5.7}$	3/6	$10^{0.8 - 4.0}$	100%	2/6	0/6	2/6	$10^{5.2 - 6.1}$	2/6	$10^{4.3 - 5.8}$
**Group Y**	$10^{4.0}$	$10^{4.0}$	1/6	0/6	4/6	$10^{2.0 - 3.7}$	3/6	$10^{0.8 - 1.0}$	100%	0/6	0/6	1/6	$10^{2.2}$	0/6	-
**Group C[e]**	$10^{4.0}$	-	2/6	0/6	5/6	$10^{0.4 - 4.1}$	4/6	$10^{1.0 - 4.0}$	100%	2/6	0/6	3/6	$10^{1.6 - 3.6}$	3/6	$10^{0.8 - 4.3}$
**Group E**	$10^{4.5}$	-			4/6	$10^{1.7 - 3.8}$	3/6	$10^{1.3 - 2.8}$	-	-	-	-	-	-	-
**Group AA**	-	-	-	-	-	-	-	-	0%	3/3	3/3	3/3	$10^{3.7 - 8.7}$	3/3	$10^{2.8 - 7.8}$
**Group F**	-	-	-	-	-	-	-	-	0%	3/3	3/3	3/3	$10^{3.2 - 8.4}$	3/3	$10^{4.0 - 8.3}$

[a] Signs refer to temperature and other clinical signs observed throughout the study including lethargy and loss of appetite. [b] GC/mL refers to genome copies per millilitre blood as measured by qPCR. . [c] Viremia pre-challenge refers to the detection of infectious virus used for immunisation. This virus expresses tdTomato and is non-haemadsorbing. [d] Viremia post-challenge refers to detection of Georgia 2007/1 challenge virus. This does not express fluorescent proteins and is haemadsorbing. [e] Group C was the prime only experimental group. [f] Prime and boost were by intramuscular immunisation. [g] The values indicate the minimum and maximum genome copies or infectious virus detected at any day pre or post challenge in any of the pigs.
